# Phosphotyrosine recognition domains: the typical, the atypical and the versatile

**DOI:** 10.1186/1478-811X-10-32

**Published:** 2012-11-07

**Authors:** Tomonori Kaneko, Rakesh Joshi, Stephan M Feller, Shawn SC Li

**Affiliations:** 1 Department of Biochemistry and the Siebens-Drake Medical Research Institute, Schulich School of Medicine and Dentistry, University of Western Ontario, London, Ontario, N6A 5C1, Canada; 2 Biological Systems Architecture Group, Weatherall Institute of Molecular Medicine, Department of Oncology, University of Oxford, John Radcliffe Hospital, Headley Way, Oxford, OX3 9DS, UK

**Keywords:** Posttranslational modification, Phosphotyrosine signaling, Ligand recognition specificity, Cancer therapeutics, Signaling circuit

## Abstract

SH2 domains are long known prominent players in the field of phosphotyrosine recognition within signaling protein networks. However, over the years they have been joined by an increasing number of other protein domain families that can, at least with some of their members, also recognise pTyr residues in a sequence-specific context. This superfamily of pTyr recognition modules, which includes substantial fractions of the PTB domains, as well as much smaller, or even single member fractions like the HYB domain, the PKCδ and PKCθ C2 domains and RKIP, represents a fascinating, medically relevant and hence intensely studied part of the cellular signaling architecture of metazoans. Protein tyrosine phosphorylation clearly serves a plethora of functions and pTyr recognition domains are used in a similarly wide range of interaction modes, which encompass, for example, partner protein switching, tandem recognition functionalities and the interaction with catalytically active protein domains. If looked upon closely enough, virtually no pTyr recognition and regulation event is an exact mirror image of another one in the same cell. Thus, the more we learn about the biology and ultrastructural details of pTyr recognition domains, the more does it become apparent that nature cleverly combines and varies a few basic principles to generate a sheer endless number of sophisticated and highly effective recognition/regulation events that are, under normal conditions, elegantly orchestrated in time and space. This knowledge is also valuable when exploring pTyr reader domains as diagnostic tools, drug targets or therapeutic reagents to combat human diseases.

## Background

### Phosphotyrosine signaling

Intracellular communication is transmitted via networks of molecules that execute information transfer using protein-mediated interactions. Post-translational modifications (PTMs) such as protein phosphorylation, acetylation, methylation and ubiquitination confer spatiotemporal dynamics to cell signaling [1]. Among these PTMs, the tyrosine phosphorylation signaling system in eukaryotes, especially in mammalian species, has been extensively studied owing to its importance in numerous cellular functions including differentiation, proliferation, motility and apoptosis as well as its therapeutic potential. In particular, mutations and aberrant expression of kinases are frequently associated with tumourigenesis [2,3].

Signaling proteins often possess a cassette-like architecture made up of catalytic domains and/or protein interaction modules [4]. One important group of protein-protein interaction modules are the autonomous domains that recognize phosphorylated tyrosine (pTyr) residues at specific sites on their target molecules [5]. These pTyr-binding protein modules and their targets are a part of an elaborate pTyr signaling system that consists of three major components that help relay molecular messages [6]. The pTyr signaling system is activated when a stimulus reaches catalytic proteins that act as “writers” of phosphorylation, the protein tyrosine kinases (PTKs). Most PTKs are phosphorylated on themselves to attain an active state, and subsequently phosphorylate other substrate proteins. A second group of proteins that contain modular domains are capable of recognizing or “reading” this phospho-modification information and thereby linking the kinase signal to downstream molecules. The phosphorylation can be subsequently “erased” by a third group of proteins, the protein tyrosine phosphatases (PTPs), therefore terminating the signal [7,8].

The human genome harbours 90 PTKs [9], hundreds of pTyr-recognition domains that include 121 members of the Src homology 2 (SH2) domain family [10-12], and more than 10,000 tyrosine phosphorylation sites [12,13]. These signaling components form an enormous network of pTyr signaling that is both robust and dynamic. Nature is equipped with multiple strategies to reduce possible misfiring of pTyr signals due to the complexity of pTyr signaling network. First, PTKs can remain inactive until they are stimulated by a proper cue, most typically by association of a specific ligand molecule to the PTK. For example, receptor tyrosine kinases (RTKs), transmembrane proteins consisting of an extracellular ligand binding site and an intracellular tyrosine kinase domain, are designed inactive until a ligand binds to the extracellular site of the RTK, which often induces RTK oligomerization [14]. Structural studies have also revealed the presence of both active and inactive conformations for many cytoplasmic and receptor kinases [15,16]. Moreover, the level of PTP activity is very high in cells, thereby ensuring that pTyr sites can be rapidly dephosphorylated [17].

Second, each component of the pTyr signaling circuit, i.e., a PTK, PTP or pTyr-binding module, possesses substrate or ligand recognition specificity to narrow down potential interaction partners [18-23]. Since all three components are the modules that bind to linear motif peptides, the specificity at the molecular or atomic level is defined by the ligand peptide sequence and can also be contributed by conformation of the peptide. The interaction specificity is further enhanced by spatiotemporal regulation of the network components, including tissue-specific or cell cycle-dependent protein expression, protein localization to subcellular compartments or a scaffold protein, and protein inactivation involving receptor internalization (endocytosis) and/or protein degradation. These multiple layers of regulatory mechanisms are essential for coordinating such a complex functional network [1,15,24,25]. However, an unintended activation of pTyr signaling, or misfiring, may occur when a circuit component malfunctions, most commonly due to mutations, overexpression or loss of a component or an element, as we will discuss some cases below.

This review will focus on the pTyr "reader" proteins contributing to this complex system. In addition to the SH2 and phosphotyrosine-binding (PTB) domains, the two archetype domain families known for pTyr binding, recent studies have shown that at least a handful of additional protein modules are capable of reading out the tyrosine phosphorylation. Here we review and explore the structure, function, specificity and therapeutic potentials of a number of typical and atypical members of the superfamily of pTyr-binding protein modules.

### The SH2 domain

The Src homology 2 (SH2) domains, a non-catalytic module containing ~100 amino acids, was first discovered by insertion-mutation analysis of the *vfps*/*fes* oncogene from the Fujinami sarcoma virus [26] (for historical perspectives of tyrosine phosphorylation studies, refer to reviews by Pawson [27] and Hunter [3]). Soon thereafter, the SH2 domain was identified in oncogenes such as *vcrk* and in the endogenous cytoplasmic proteins phospholipase Cγ1 (PLCγ1) and the Ras GTPase activating protein (RasGAP) [28,29]. SH2 domains have since been identified in a wide range of eukaryotic species, including yeast, but primarily in metazoans [7,11]. A recent tally finds 121 SH2 in 111 proteins in the human genome [11]. Proteins containing SH2 domains include those that function as kinases, adaptors, phosphatases, ubiquitin ligases, transcription factors, guanine nucleotide exchange factors and phospholipid-based secondary signaling molecules [5,12,30]. Studies in almost two decades have demonstrated the tyrosine phosphorylation-dependent nature of typical SH2 domain-ligand interactions [31-34], the central role played by SH2 domains in connecting activated receptor tyrosine kinases, such as the epithelial growth factor receptor (EGFR) and the platelet-derived growth factor receptor (PDGFR), to cytoplasmic signaling molecules [29,35]. In addition, kinase SH2 domains are essential in regulating the catalytic activity of cytoplasmic kinases as exemplified for the Src family as well as the Fes and Abl kinases [15]. A growing picture illustrates that kinase SH2 domains may regulate catalytic activity utilizing diverse mechanisms [16,36]. These and other lines of work establish the SH2 domain as a key player in the cellular signaling system in a pTyr-dependent manner [37].

#### Architecture of the SH2 domain

**Figure 1:**
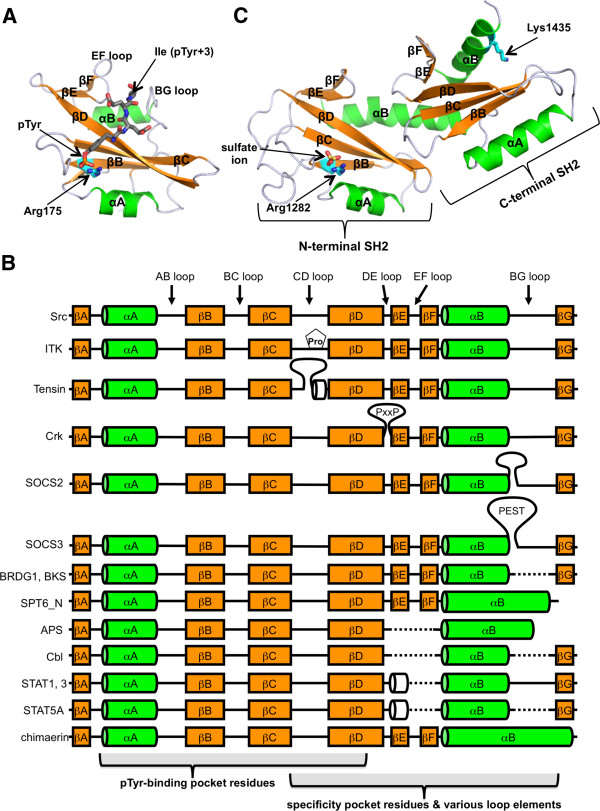
***Structure and sequence patterns of the SH2 domain.*** (**A**) Structure of the v-Src SH2 domain in complex with the pYEEI peptide (PDB ID: 1SPS). The two conserved α-helices are coloured green, and the seven β-strands are coloured orange. The peptide is shown as grey sticks. The phosphate group of pTyr binds to Arg175 located on the βB strand of the SH2 domain. The pTyr+3 Ile side chain is captured by a hydrophobic pocket provided between the EF and BG loops. (**B**) Conservation and variation in the secondary structural elements of SH2 domains based on experimentally determined structures. Refer to [40] for a list of SH2 domain structures. The N-terminal half of an SH2 domain is dedicated to pTyr recognition and is much less variable than the C-terminal half where the specificity pocket is located. A dashed line indicates that the element does not exist in an SH2 domain. Structural variations are observed more often in the C-terminal half. For example, the BG loop of the STAP family (BRDG1 & BKS) and the Cbl family SH2 domains are much shorter than in other SH2 domains, which results in an open pocket capable of binding a hydrophobic pTyr +4 residue [40,43]. Pro287 of the ITK SH2 domain is susceptible to *cistrans* isomerization via its CD loop, which leads to a switch of binding partners [44-46]. The long, proline-rich DE loop insertion in the Crk SH2 domain is the binding site for the Abl SH3 domain [47]. (**C**) The tandem SH2 domains of the transcription factor Spt6. Four research groups have reported crystal and solution structures, which are essentially identical to each other [48-51]. Shown here is the crystal structure of the *Saccharomyces cerevisiae* Spt6 with a sulfate ion located in the "canonical" phospho-residue binding pocket of the N-terminal SH2 domain (PDB ID: 3PSK) [48]. Mutagenesis studies and NMR titration analysis suggested that this pocket, involving Arg1282, as well as a positively charged patch, including Lys1435 of the C-terminal SH2 domain (residues shown as cyan sticks), are the binding sites of the phosphorylated CTD peptides [49,50,52].

As represented by the v-Src SH2 domain (Figure 1A), the structure of an SH2 domain features two α-helices (αA and αB) sandwiching a β-sheet consisting of seven anti-parallel strands (βA-βG) [38,39]. Based on the experimentally determined structures of ~ 70 unique SH2 domains in the Protein Data Bank (PDB), the N-terminal region of the SH2 domain that provides a pTyr-binding pocket is more conserved than the C-terminal half of the SH2 domain that exhibits greater structural variability (Figure 1B). For instance, sequence deletion or insertion is found primarily in the βE-βF and BG loop regions (Figure 1B). In addition, structure-based sequence alignment revealed that the most conserved residues are clustered on the βB strand [40]. For the majority of experimentally solved SH2-ligand complex structures, the bound pTyr-peptide forms an extended conformation and binds perpendicular to the central β-strands of the SH2 domain (Figure 1A). Specific residues in the N-terminal region (αA to βD) form a positively charged pocket for binding of the pTyr residue [38,39,41]. A conserved arginine residue on the strand βB (Arg175 in the v-Src SH2 domain) (Figure 1A) plays the central role in forming bi-dentate hydrogen bonds with the phosphate moiety of pTyr. In contrast, a hydrophobic pocket provided by the second half of the domain (i.e. βD to βG) engages residues C-terminal to the pTyr of a ligand peptide to confer specificity [12,30,42]. 

#### Specificity of the SH2 domain

SH2 domains are equipped for the specific recognition of a subset of pTyr–containing ligands [20,22,53-55]. A number of studies have established that binding affinity of an SH2 domain to a pTyr-containing ligand is moderate, with the typical affinity range between 0.1 μM and 10 μM for equilibrium dissociation constant values (*K*_D_) [56-60]. This moderate affinity is considered to be crucial for allowing transient association and dissociation events in cell signaling. Indeed, artificially increased affinity using an engineered SH2 domain (called the pTyr superbinder) has been shown to cause detrimental consequences to cells [61]. While the pTyr-binding pocket, which is present in the N-terminal half and highly conserved in the SH2 domain, provides the basal affinity for ligand binding with approximately a half of the total binding free energy [62], the hydrophobic pocket present in the C-terminal half of an SH2 domain provides specificity towards a hydrophobic residue in a peptide ligand. Recent studies suggest that the major positional specificity of an SH2 domain is conferred by the EF and BG loops which regulate ligand access to specificity pockets in an SH2 domain. Thus, distinct loop composition and configuration determines whether an SH2 domain has specificity for a residue at the second, third or fourth position C-terminal to the pTyr residue [10,11,20,40,63,64]. The wealth of experimentally solved SH2 domain-ligand complex structures allows the systematic ultrastructural investigation into how variations in the specificity pocket leads to distinct specificities with bioinformatics tools. Meanwhile, additional subtlety and sophistication in pTyr-peptide discrimination have been demonstrated by a recent study highlighting the importance of permissive and non-permissive residues proximal to pTyr in the ligand sequence [65]. The study provided evidence that local sequence context provides an additional layer of specificity enhancement beyond the general sequence motifs uncovered by regular degenerate peptide library screens [20,66].

#### The Spt6 SH2 domain: a common ancestor of pTyr recognition?

The yeast genome encodes only one SH2 domain-containing protein, the transcription factor Spt6 [7]. The C-terminal region of the protein, initially predicted to contain a single SH2 domain, binds to the C-terminal domain (CTD) of the RNA polymerase II [52]. The CTD consists of an abundance of repeats (52 in human, 26 in yeast) of the heptad sequence YSPTSPS, in which each tyrosine, serine, and threonine residues can be phosphorylated. Furthermore, prolines are subjected to *cistrans* isomerization, adding another layer of complexity and dynamics to the CTD [67,68]. The SH2 domain of Spt6 has been considered a prototypical SH2 domain for several reasons. (I) Spt6 is present in yeast that does not contain a PTK, (II) Yoh et al. demonstrated that the Spt6 SH2 domain region binds to Ser-phosphorylated CTD, and (III) Spt6 is conserved in eukaryotes including slime moulds and plants [7,11,52,69]. Indeed, the recently solved structures of the C-terminal region of Spt6 revealed that the region actually contains two SH2 domains in tandem that are intimately associated with each other (Figure 1C) [48-51]. The phospho-binding pocket of the N-terminal SH2 domain, which contributes to CTD phosphopeptide binding, contains an arginine that is invariant among eukaryotic SH2 domains [49,50,52]. In contrast, the corresponding pocket in the C-terminal SH2 domain lacks an arginine, and NMR titration studies suggest that this pocket is not used for peptide binding. Instead, a positively charged patch on the surface of the C-terminal SH2 domain participates in CTD binding (Figure 1C) [49,50]. Interestingly, the tandem SH2 domains have shown low affinities (with dissociation constants in the millimolar range) for both pTyr- and pSer-containing peptides derived from the CTD [50]. It is proposed that the binding of the Spt6 tandem SH2 domains to the polymerase may be significantly enhanced *in vivo* as the CTD contains numerous repeats of the phosphorylated heptad sequence that can increase the effective local concentration of the binding target for the tandem SH2 domains [50]. From an evolutionary standpoint, it is likely that the Spt6 SH2 domains provided the prototype for a family of modular domain for the phospho-specific interaction that have later evolved to be specific for phosphotyrosine. Notwithstanding this viewpoint, SH2 domains have been identified in abundance in protozoans such as choanoflagellates [7,8,70].

#### Atypical ligand recognition modes

Although SH2 domains are the largest group of pTyr-binding modules [5,10], it has been shown that certain SH2 domains have the ability to bind ligands in a tyrosine phosphorylation-independent manner [10,34,71-78]. For example, the SH2 domains of tensins and SAP bind to both phosphorylated and non-phosphorylated forms of ligand peptides [73-75,78,79]. Some SH2 domains feature a secondary site located outside of the primary ligand-binding site to engage a pTyr-ligand protein using two sites [76,80]. In this regard, Anderson and colleagues identified a second binding site on the C-terminal SH2 domain of the phosphoinositide 3-kinase p85α subunit that binds the Raf family kinase member A-Raf in a phosphorylation-independent manner [80]. The authors posit that the second binding site increases the target selectivity of the SH2 domain. Biochemical and structural analysis illustrated pTyr-independent interaction between the N-terminal SH2 domain of PLCγ1 and the tyrosine kinase domain of the fibroblast growth factor receptor (FGFR) via a secondary binding site, in addition to the canonical pTyr-binding primary site [76]. In another scheme, an SH2 domain may contain sequence motifs that are recognized by distinct types of modular domains. A long, proline-rich insertion into the DE loop of the Crk SH2 domain (Figure 1B) is recognized by the Src homolgy 3 (SH3) domain of the Abl kinase [47]. Sequence analysis of Crk orthologs suggests that this insertion is of recent evolutionary origin as it is identified only in mammalian species [11]. This provides an example of loop evolution that enriches the pTyr signaling network by introducing a novel interaction.

#### Interplay between SH2 and kinase domains

Among the 90 PTKs in the human genome, 32 are cytoplasmic tyrosine kinases [81]. Notably, 28 of them also contain an SH2 domain in tandem with the kinase domain (with the exceptions of TNK1, ACK, FAK and Pyk2). This suggests a strong physical-functional relationship between the kinase and SH2 domains. Mayer et al. demonstrated that a kinase-associated SH2 domain promotes phosphorylation of substrates, which they termed processive phosphorylation [82]. In the course of processive phosphorylation, the kinase domain of a cytoplasmic tyrosine kinase phosphorylates a substrate on the tyrosine site, that is then tightly bound by the SH2 domain, allowing the associated kinase domain to carry out further phosphorylation of the substrate (or a second substrate molecule associated with the first substrate) [19,83]. Thus the assembly of SH2-kinase domain cassettes allows the physical association required for function of the two domains [4,5,15]. Moreover, recent bioinformatics analysis of protein sequences for 330 *bona fide* SH2-binding motifs revealed that tyrosine phosphorylation sites in human proteome are significantly enriched in the vicinity of the SH2 domain-binding sites [84], which may indicate that processive phosphorylation is a rather common phenomenon. Processive phosphorylation is perhaps unique to the SH2-kinase domain combination, because the PTB domain (see below) does not coexist with a tyrosine kinase domain in any human protein [85]. As we discuss later, direct intramolecular interaction between the kinase and SH2 domains is essential for activity in some PTKs, which represents another physical and functional interaction between the two domains and a potential target for treatment in cancer cells.

#### Multifunctional loops for the SHP SH2 domains

**Figure 2:**
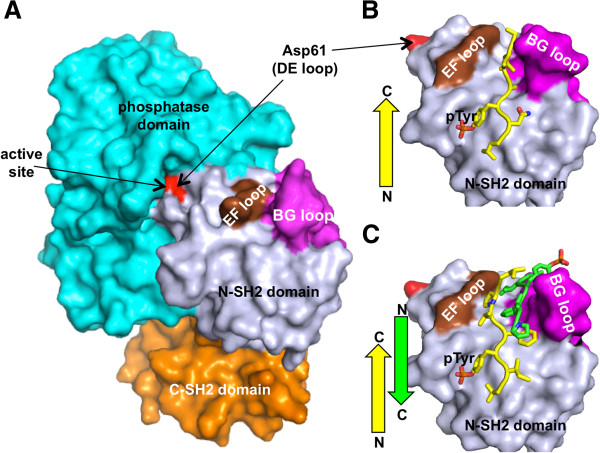
***Surface loops in the SH2 domain confer multiple binding modes to the tyrosine phosphatase SHP2.*** The N-terminal SH2 (N-SH2), C-terminal SH2 (C-SH2) and the phosphatase domains are coloured in light blue, orange, and cyan, respectively. The EF and BG loops of the N-SH2 domain are coloured brown and magenta, respectively. Molecular orientation is aligned for the N-SH2 domain, and drawn to scale. (**A**) The inhibitory state of SHP2 (PDB ID: 2SHP) [87]. The DE loop region of the N-SH2 domain, including the side chain of Asp61 (coloured red), mimics a pTyr substrate and blocks the active site of the phosphatase domain. In this conformation, the BG loop contacts the EF loop and inhibits ligand binding. (**B**) The 1:1 binding mode (PDB ID: 3TL0) [90]. The bound LNpYAQLW peptide is coloured yellow. The C-terminal region of the single peptide binds to a cleft between the EF and BG loops. (**C**) The 1:2 binding mode, in which the two identical peptides, with a sequence VIpYFVPL, form a short, antiparallel β-sheet and bind to a single SH2 domain (PDB ID: 3TKZ) [90]. The BG loop is positioned to accommodate the two peptides.

Interplay between an SH2 domain and a catalytic domain has also been observed for phosphatases. Pei et al. reported that the SH2 domains of tyrosine phosphatase SHP-1 regulates its catalytic activity via an auto-inhibition mechanism [86]. Since then, structural studies of SHP-1, and its paralog phosphatase SHP-2, have revealed multiple conformations for the SHP phosphatases. The first crystal structure of SHP-2 demonstrated its inactive conformation, in which Asp61 of the DE loop from the N-terminal SH2 domain mimics pTyr moiety and directly blocks the catalytic pocket of the phosphatase domain (Figure 2A) [87]. Interestingly, in this inactive conformation, the cleft between the EF and BG loops of the N-SH2 domain is closed and ligand binding is disabled. A similar inhibitory conformation was also observed for SHP-1 [88]. A recent model proposed that activation of the SHP-1 phosphatase requires binding of a pTyr ligand to the SH2 domains and a subsequent large structural rearrangement of the C-terminal SH2 domain to allow dissociation of the N-terminal SH2 domain from the catalytic pocket [89]. A further structural study illustrated that the N-terminal SH2 domain of SHP-2 possesses the ability to act either in the single-peptide or in the double-peptide binding mode, depending on the peptide sequence [90]. The single-peptide binding mode follows a canonical ligand binding mechanism, i.e., binding to an open cleft between the EF and BG loops (Figure 2B). In the double-peptide binding mode, one of the peptides binds the canonical pocket in a pTyr-dependent manner whereas the other pairs up with the first peptide to form a short antiparallel β-sheet (Figure 2C). The authors propose that such a property of the SH2-dual peptide interaction suggests the SH2 domain may serve as a scaffold for two ligand molecules. 

#### Phosphorylation-dependent binding partner switching

**Figure 3:**
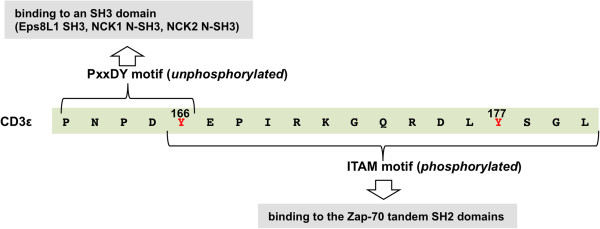
***Binding partner switch induced by tyrosine phosphorylation.*** The sequence of the T-cell receptor subunit, CD3ε, contains two interaction motifs overlapping at a tyrosine phosphorylation site. The two phosphorylation sites Tyr166 and 177 are coloured red. The binding partners of the motifs, depending on the phosphorylation state, is schematically depicted as highlighted boxes.

Post-translational modification such as tyrosine phosphorylation may act as a switch for some proteins. For instance, the presence or absence of phosphorylation may provide a mechanism for alternative binding to distinct protein partners [84]. This type of regulation is underscored in the multifaceted interaction between the T-cell receptor subunit CD3ε and either the ZAP-70 tandem SH2 domains or the Eps8L1 and the N-terminal NCK SH3 domains (Figure 3) [91-93]. CD3ε harbours both an immunoreceptor tyrosine-based activation motif (ITAM) and a PxxDY motif. These two motifs overlap at Tyr166, which may be phosphorylated (Figure 3). While phosphorylation of Tyr166, along with Tyr177 in the ITAM motif, promotes the binding of the tandem ZAP-70 SH2 domains, the phosphorylation of Tyr166 also abrogates SH3 domain binding via the PNPDY motif (Figure 3) [91-93]. Bioinformatics analysis suggests that there exist a plethora of such phosphorylation sites on signaling proteins which could act as regulatory switches for selective protein-protein interactions [84]. Moreover, phosphorylation-dependent partner switching has been documented to also occur on other modular domain-mediated interactions, as elucidated below for the interaction between E-cadherin and the PTB and HYB domains [94-96]. 

### The PTB domain

The phosphotyrosine-binding (PTB) or phosphotyrosine-interacting domain, first identified in the adaptor protein Shc [97-99], is the second largest family of pTyr-binding modules. Approximately 60 human proteins contain a PTB domain [85]. Although none have been identified from yeast or plants so far, two PTB domains have been found in the slime mould *Dictyostelium discoideum*[85], and 31 in the choanoflagellate *Monosiga brevicollis*[100]. The proteins harbouring a PTB domain strictly act as adaptors or molecular scaffolds [101], with possible exceptions at least in *M*. *brevicollis*, where multiple tyrosine kinases that contain a PTB domain have been identified [6]. Indeed, biochemical studies indicate that the PTB domain of the *M*. *brevicollis* tyrosine kinase HMTK1 assists in targeting of a pTyr-containing substrate peptide [102]. While a third of all PTB-containing proteins identified contain a single copy of the domain, the remaining two-thirds feature a PTB domain occurring in combination with other modular domains such as SH2, SH3, PDZ or SAM [85]. PTB domain-containing proteins are involved in a host of signaling processes, including those involving receptor tyrosine kinases, cytokines and lipoprotein receptors, and cellular functions such as cell division and cell-cell adhesion [101].

#### Architecture of the PTB domain

**Figure 4:**
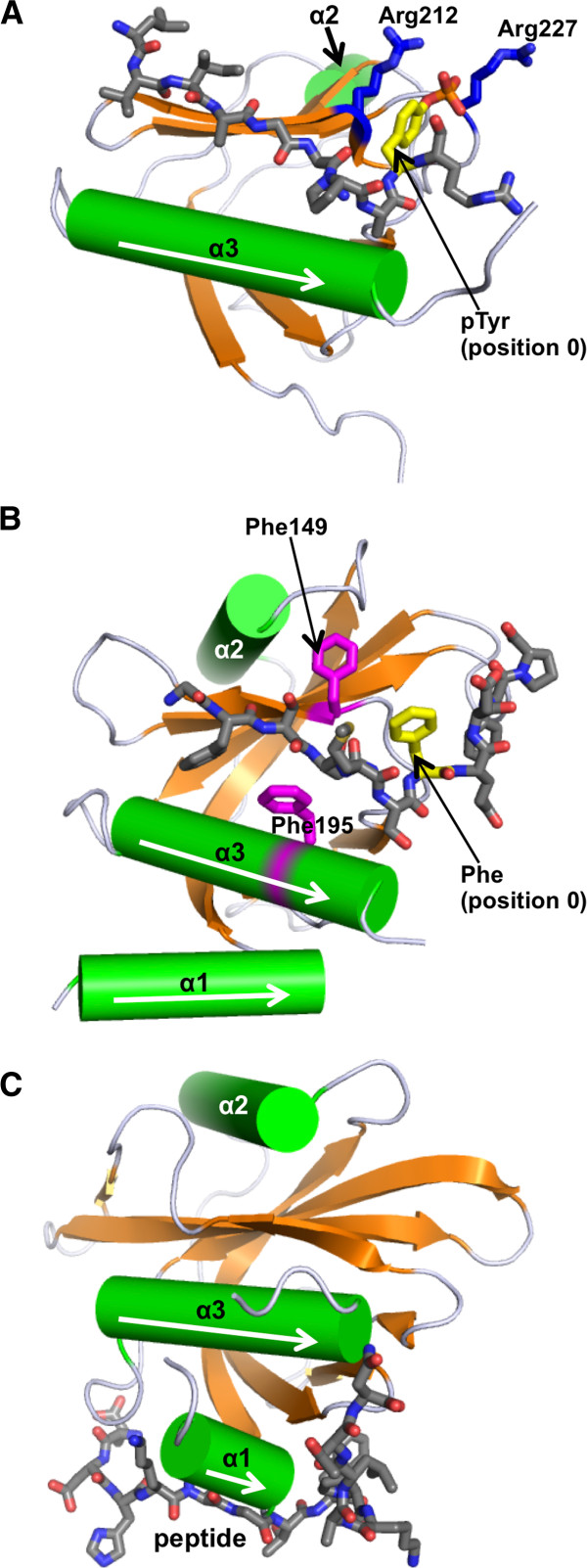
***Diversity in ligand recognition in the PTB domain family*.** PTB domains are shown in ribbon representations, with α-helices in green, and β-strands in orange. Bound peptides are drawn as gray sticks. (**A**) The PTB domain of IRS-1 bound to a pTyr-containing peptide derived from interleukin 4, containing an NPApY sequence (PDB ID: 1IRS) [105]. The two arginine residues, Arg212 and Arg227 (coloured blue), provide electrostatic contacts with pTyr at position 0 (coloured yellow). (**B**) The Numb PTB domain bound to an NAK-derived peptide (PDB ID: 1DDM) [111]. The peptide contains an NMSF sequence, but not a tyrosine. Phe149 and Phe195 (coloured magenta) of the PTB domain are essential for peptide binding. (**C**) The tensin2 PTB domain bound to a peptide derived from DLC-1 (PDB ID: 2LOZ) [112]. The peptide, which does not contain an NXX[Y/F] motif, binds to a novel site on the PTB domain that involves the α1 helix.

Johnson and colleagues divided the PTB domain family into three classes, namely Shc-like, IRS-like and Dab-like, based on the domain structure [85]. Although, ligand recognition by the Shc-like and IRS-like PTB domains is considered to be tyrosine phosphorylation-dependent, the majority of the remaining PTB domains, classified as the Dab-like domains, are phosphorylation-independent in ligand binding [85]. Structural analysis has revealed that the PTB domains are characterized with the pleckstrin homology (PH) domain “superfold”, although they share little sequence identity with PH domains [103]. All PTB domains encompass a minimal fold containing two orthogonally arranged β-sheets composed of seven anti-parallel β-strands (Figure 4A). The β-sheets pack against two α-helices, α2 and α3 (nomenclature of the secondary structures follows that defined in [104]). The Shc-like and Dab-1-like PTB domains have an additional N-terminal helix (α1 helix), whereas the IRS-1-like domains have an extremely truncated α2 helix, which represents the minimal domain fold (Figure 4A) [105-109]. The PTB domains bind peptide ligands in an L-shaped hydrophobic groove contoured by the β5 strand and the α3 helix [110]. The peptide usually docks with its N-terminal residues forming an anti-parallel β-strand and the C-terminal of the peptide arranged as a type I β-turn. 

#### Tyrosine phosphorylation-dependent and independent ligand binding

Similar to the SH2 domain, PTB domains may bind tyrosine phosphorylation sites in cellular proteins. Proteomic studies have revealed that the two domain families may in fact target overlapping pTyr sites with micromolar affinities [57-59,113]. However, unlike SH2 domains, the specificity of a PTB domain is primarily focused towards amino acids N-terminal to the pTyr residue in a peptide, most commonly in an NPXpY or NPXY sequence motif [114]. Moreover, the majority of PTB domains prefer a non-phosphorylated tyrosine residue. In fact, phosphorylation of a peptide is inhibitory to binding to some members of the Dab-1-like PTB domain group [85]. Interestingly, a number of PTB domains can bind the head groups of inositol phosphates with varying affinities, a function observed also in some of the structurally similar PH domains [85,110]. PTB domains appear to commonly bind to phospholipids through a patch of basic residues on the surface of the domain, although the actual residues that bind to the phospholipid are variable or not resolved for most PTB domains [85,103]. Interestingly, a phosphopeptide and a phospholipid can compete against each other in binding to a PTB domain, as has been shown for the Shc PTB domain [115].

#### The versatile Numb PTB domain

Numb is an adaptor and endocytic protein that plays an important role in asymmetric cell division and embryogenesis [116]. It contains a Dab-1-like PTB domain indispensable for its function. Biochemical and structural studies have suggested that the Numb PTB domain is capable of binding to either non-phosphorylated sequences that contain an NXX[Y/F] motif or pTyr-containing sequences [104,111,116,117]. Structural analysis unraveled the molecular basis of promiscuous binding by the Numb PTB domain to peptides that possess distinct primary and secondary structures [104,111]. Figure 4B shows the complex structure of the Numb PTB domain in complex with a non-phosphorylated peptide that contains the sequence NMSF derived from the Numb-associated kinase (NAK) [111]. Interestingly, mutation of the sequence from NMSF to NAAF resulted in 15-fold increase in binding affinity, implying that the physiological association between the Numb PTB domain and NAK may not be optimized for high affinity [111]. Moreover, the Numb PTB domain provides a binding site for another domain in an isoform-specific manner. The p72 and p66 isoforms of Numb contain an 11-residue insert within the PTB domain after the α2 helix, which serves as a binding site for the PDZ domain of LNX (Ligand-of-Numb), an E3 ubiquitin ligase required for isoform-specific Numb degradation [118]. Recently, we found that Numb serves as a new player in epithelial to mesenchymal transition, a critical step in cancer progression and metastasis [94,119]. The Numb PTB domain binds to the N^751^VYYY motif located in the cytoplasmic region of E-cadherin, but phosphorylation of the motif by Src results in dissociation of the PTB domain, suggesting that Src activation negatively regulates the interaction between Numb and E-cadherin [94].

#### The tensin family SH2 and PTB domains

Unlike SH2 domains, which often coexist with a kinase or phosphatase domain in a protein, the human PTB domain-containing proteins invariably lack a catalytic domain, with the single exception of the presence of a PTP-like domain in the tensin family (see a later section for detail) [85,120,121]. Tensins 1–4 are focal adhesion proteins that contain an SH2 domain and a PTB domain in tandem [122]. Similar to the tensin-like lipid and tyrosine phosphatase PTEN [123], tensins 1–3 have been identified as tumour suppressors. Tensins interact with another tumour suppressor, deleted in liver cancer 1 (DLC1), and suppress focal adhesions and cell migration in various cancers [122,124]. The SH2 domain of tensin3 is itself phosphorylated by the Src kinase [125]. Lowy and colleagues suggest that tyrosine phosphorylation on the tensin3 SH2 domain provides a mechanism for controlling ligand binding and that phosphorylation of the SH2 domain by the Src kinase endows tensin3 with proto-oncogenic properties [125]. The study also indicated that binding to DLC1 was not dependent on tyrosine phosphorylation of the SH2 domain of tensin3, whereas binding to some other ligands such as the focal adhesion kinase was enhanced by the SH2 phosphorylation. Another study showed that the SH2 of tensin2 has the ability to bind non-phosphorylated DLC1 [78]. These two observations indicate that the phosphorylation state of the ligand proteins as well as that of the SH2 domain can regulate SH2-ligand interactions in tensins [78,125]. Moreover, the PTB domain of tensin2 displays a novel peptide binding mode. Although it was observed that the tensin1 PTB domain can bind an NPXY peptide in a canonical manner [126], as well as a DLC1 peptide via a yet uncharacterized mode [75], the tensin2 PTB domain, which can also bind the NPXY motif [127], has been determined to utilize its N-terminal helix (α1) to engage a non-NPXY site in DLC1 [112]. The binding surface in the latter PTB domain is opposite to the canonical NPXY peptide binding site and the peptide ligand adopts an elongated conformation rather than the conventional β-turn structure (Figure 4C) [112]. These observations indicate the presence of peptide binding surface specializations among the tensin family PTB domains.

### Atypical pTyr recognition domains and proteins

#### The HYB domain

**Figure 5:**
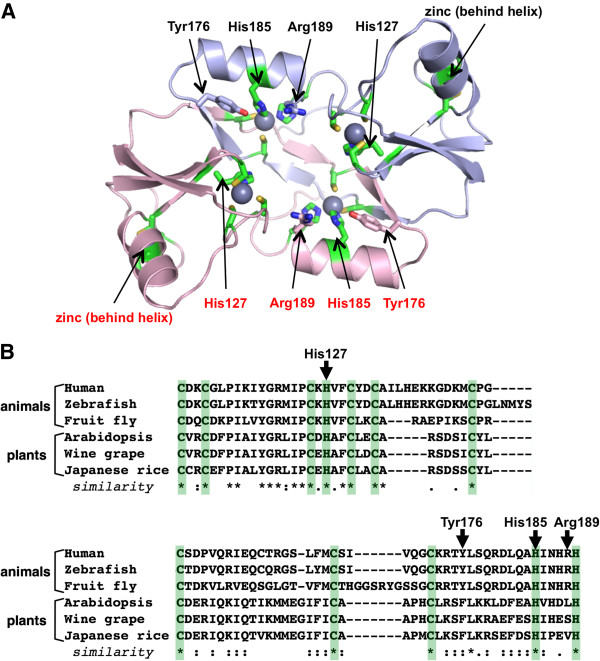
***The structure and sequence conservation of the HYB domain.*** (**A**) The HYB domain is coordinated with six zinc ions. Shown is the homo-dimeric structure of the Hakai HYB domain (showing the two chains with different colours) (PDB ID: 3VK6) [96]. Zinc ions are depicted as spheres. The 24 residues (12 per monomer) coordinating the zinc ions are shown as green sticks. The four residues, His127, Tyr176, His185, and Arg189 from each monomer, identified as sticks, mainly contribute to pTyr binding by providing a positively charged pocket. (**B**) HYB domain-like sequences identified in animals and plants. The alignment was generated by the program MAFFT [130]. The 12 zinc-binding residues are shaded green, showing that all of them are strictly conserved within these species. The human Hakai HYB domain was aligned with following sequences with UniProt IDs: zebrafish (Q5RGV5), fruit fly (Q9VIT1), *Arabidopsis thaliana* (Q9LFC0), wine grape (F6HKX7), and Japanese rice (Q0IWQ6).

Hakai, a protein that binds to E-cadherin in a tyrosine phosphorylation-dependent manner, serves as an E3 ubiquitin ligase for the latter and induces endocytosis and degradation of E-cadherin [95]. Hakai is also known to interact with an RNA-binding protein, the PTB-associated splicing factor, that targets mRNAs encoding cancer-related proteins [128]. Hakai resembles the E3 ligase c-Cbl in that they both harbour a pTyr binding domain (an SH2 domain in the case of c-Cbl), a region homologous to the RING finger motif and a proline-rich region [95]. The pTyr binding region in Hakai was initially thought to be an SH2 domain, but this assumption was proven to be incorrect by recent studies showing that a unique, zinc co-ordinated domain formed by the dimerization of two Hakai monomers is responsible for pTyr recognition [95,96]. This novel pTyr recognition domain, now named the HYB domain, is formed via the dimerization of a 100-residue stretch, including the RING finger motif, in an anti-parallel orientation (Figure 5A). The dimer coordinates six zinc ions with a total of 24 Cys or His residues (four residues per zinc ion; Figure 5A). Crystallographic and NMR titration experiments revealed that the positively charged pTyr-binding pocket, which is responsible for binding phosphorylated E-cadherin, is situated in the interface of the dimer. In particular, phosphorylation of the N^751^VYYY motif on the Tyr residues and acidic residues flanking the motif are necessary for binding to the HYB domain, with a *K*_D_ value of 7.2 μM for the N^751^VYpYY containing E-cadherin peptide [96]. Because this motif also mediates the binding to the Numb PTB domain when it is not phosphorylated [94], it provides yet another example of the tyrosine phosphorylation-dependent binding partner switch. Interestingly, the HYB domain has also been identified in the testis-specific ubiquitin ligase ZNF645, but its target specificity is apparently distinct from that of the Hakai HYB domain [96]. Moreover, sequence inspection suggests that the Numb-associated E3 ligase LNX may contain an HYB domain [96]. Thus, the HYB domain appears to be a recurring feature in certain E3 ligases and its presence in cell adhesion regulatory proteins such as Hakai suggests a role for this domain in cell-cell adhesion and cancer metastasis [129]. The HYB domain is likely conserved through evolution as a sequence search identifies HYB-like domain sequences in different animals and plants with complete conservation of the zinc-coordinating residues (Figure 5B). 

#### The GEP100 PH domain

The PH domain was first described in pleckstrin, a substrate of protein kinase C (PKC) [131,132]. It has since been identified in a large number of signaling and cytoskeleton-associated proteins. By virtue of its ability to bind phospholipids, in particular inositol phosphates, the PH domain plays an important role in targeting the corresponding protein to the plasma membrane [103,133]. Interestingly, some PH domains are found to bind to proteins in a phosphorylation-dependent manner [110,133-137]. The PH domain of GEP100 (also known as BRAG2 or IQSEC1), a guanine nucleotide exchange factor for the small GTP-binding protein Arf6, binds to the EGF receptor residues pTyr1068 and pTyr1086, both of which are part of a YXNQ motif [138]. These two pTyr sites are also known to recruit the adaptor proteins Grb2 and Shc as well as the transcription factor STAT3 via their SH2 and/or PTB domains [139,140]. However, it has been shown that the GEP100 pathway may not necessarily interfere with the Grb2 pathway in breast cancer [141]. Co-expression of GEP100 and Arf6 turned non-invasive MCF7 cells to become invasive upon EGF stimulation [138,142]. Therefore, this pathway is a potential new target for breast cancer therapeutics. The crystal structure of the GEP100 PH domain alone has been solved (PDB database entry 3QWM), but a PH domain – pTyr-peptide complex structure is still missing, since synthetic pTyr1068 and 1086 peptides immobilised on a carrier membrane can directly bind to the GEP100 PH domain, but have a surprisingly low binding affinity to it in solution (S.F. et al., unpublished data). The reason for this currently remains unclear. By contrast, the native EGFR receptor is effectively precipitated by the GEP100 PH domain upon activation, i.e., when phosphorylated on pTyr1068 and/or pTyr1086.

#### The PKCδ and PKCθ C2 domains

**Figure 6:**
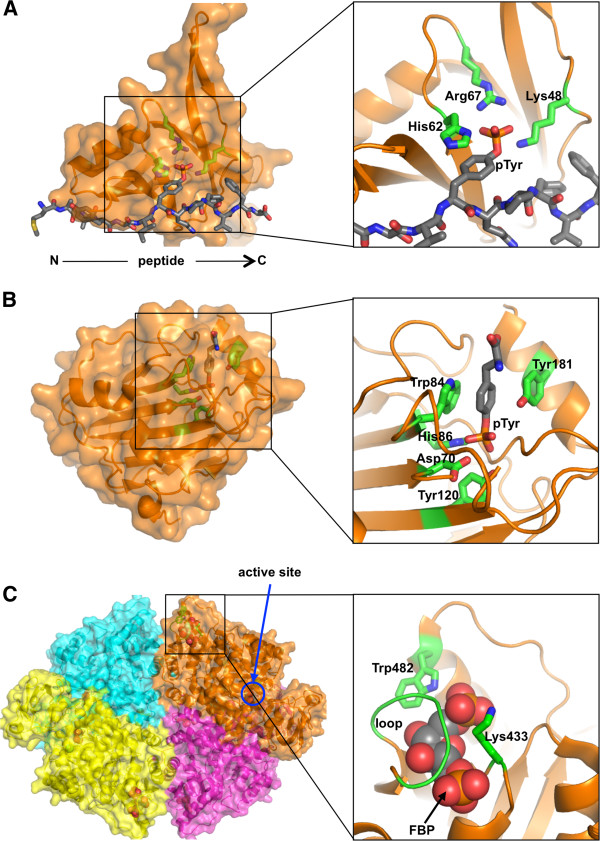
***Atypical pTyr recognition proteins*.** On the left panels, molecular surface of each protein is overlaid on ribbon representation. (**A**) The C2 domain from the human PKCδ bound to a pTyr-peptide (PDB ID: 1YRK) [145]. The peptide is shown as grey sticks. Positively charged residues (green sticks) of the C2 domain that engage the pTyr moiety are shown to highlight the histidine-phenyl ring stacking feature. (**B**) pTyr binding by the human RKIP (PDB ID: 2QYQ) [149]. The structure features a deep pocket complementary to the pTyr side chain. The structure also highlights the lack of lysine and arginine in the binding pocket. (**C**) The homo-tetrameric active form of the human PKM2 bound to FBP (PDB ID: 3BJF) [150]. Each monomer is depicted with a different colour. The allosteric activator FBP is drawn as space-filling models. The distal active site from a monomer is identified with a blue circle. In the inset, the "lip" of the FBP-binding pocket created by the Lys433 and Trp482 residues and a loop region is coloured green. A pTyr ligand also binds to this region, and promotes the release of the FBP molecule, which results in inactivation of PKM2.

The C2 domain belongs to one of the largest domain families with over 200 members in human [103,143]. This ~130 residue module primarily binds to phosphatidylserine in the cell membrane in a calcium-dependent manner. The C2 domain has a core structure of a β-sandwich formed by eight antiparallel strands (Figure 6A). The calcium binding sites are located in the inter-strand loops [103]. It has been shown that C2 domains have disparate calcium dependency for activity and a weak affinity for most phospholipids, suggesting that they may have other binding functions [103,144]. Benes et al. demonstrated that the C2 domain of the Ser/Thr kinase PKCδ can recognize a pTyr peptide derived from CDCP1 (CUB domain-containing protein 1) in a sequence-specific manner, with a *K*_D_ of 240 nM [145,146]. CDCP1, a transmembrane protein overexpressed in a number of cancers, is a substrate of the Src family kinases [147]. The C2 domain-containing PKCδ is the first example of a Ser/Thr kinase displaying a pTyr binding capability. Subsequently, the C2 domain of PKCθ, which shares 70% sequence identity with that of PKCδ [145], was also found to bind to the tyrosine-phosphorylated CDCP1 peptide with a similar affinity [148]. Importantly, this binding is the key for activation of PKCθ from an autoinhibitory state, which is mediated by an intramolecular interaction involving the C2 domain. The crystal structure of the PKCδ C2 domain in complex with an optimal pTyr peptide demonstrates that the pTyr-peptide binds in an elongated conformation across two β-sheets (Figure 6A). The C2 domain coordinates the phosphate group of pTyr in a deep pocket using positively charged lysine and arginine residues (Figure 6A). Moreover, the phenyl ring of pTyr is stabilized by a unique ring-stacking interaction with a histidine residue that is proximal to the phosphate-binding arginine residue (Figure 6A) [145]. 

#### The catalytically inactive PTP domain

**Figure 7:**
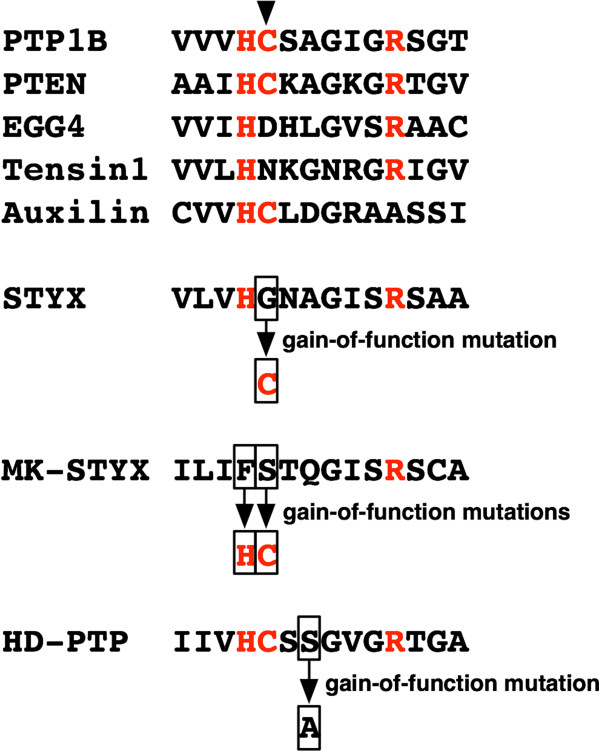
**Mutations that confer PTP activity to catalytically-dead phosphatases.** The catalytic signature motif sequences are extracted from the active phosphatases PTP1B and PTEN, along with five phosphatase-like domains. The His, Cys and Arg residues in the HCX_5_R motif, conserved among active PTP domains, are coloured red. The arrowhead indicates the catalytic cysteine. Mutations that have experimentally proven to restore catalytic activity are also indicated, for STYX [152], MK-STYX [157] and HD-PTP [159]. EGG4/5, Tensin1 and auxilin are predicted to be devoid of PTP activity owing to the lack of a signature motif residue [121,158].

The protein tyrosine phosphatase (PTP) family proteins dephosphorylate a pTyr residue of substrate proteins ("erasers" in the toolkit). There are 107 PTPs in human, of which 61 phosphatases are classified as the dual-specificity phosphatase subfamily, which also possess Ser/Thr phosphatase activity in addition to pTyr dephosphorylation [151]. The PTP family phosphatases share a conserved domain fold and possess a catalytic cysteine in the HCX_5_R motif located on the active site loop [152-154]. However, sequence analysis led to the identification of PTP domain homologs that lack the catalytic cysteine or another essential residue in the motif (Figure 7) [153,154]. Such findings were first described for the protein named STYX (phospho-serine or threonine or tyrosine interaction protein), which is similar in sequence to PTPs, except for a Cys to Gly substitution in the signature motif (Figure 7) [152,155]. This renders the STYX protein unable to catalyze dephosphorylation. However, the STYX Gly-to-Cys mutant conferred catalytic activity to the protein and the mutant demonstrated phosphatase activity for both the pTyr and pThr residues [152]. Since then, many more PTP-like domains have been identified [153,154,156-159]. For example, EGG4 and EGG5, two almost identical proteins in *Caenorhabditis elegans*, contain a PTP-like domain without a catalytic Cys residue (Figure 7). Cheng, et al. reported that the domain binds to tyrosine residues in the activation loop of a kinase that regulates the oocyte-to-embryo transition [158]. This interaction is enhanced, although not absolutely necessary, by phosphorylation of the tyrosines. Haynie and Ponting have proposed that the N-terminal regions of two proteins, tensin1 and auxilin, are PTP-like domains, in which the former lacks the catalytic cysteine, whereas the latter lacks the arginine of the HCX_5_R motif (Figure 7) [121]. Interestingly, the PTP-like domain is immediately followed by a C2 domain in both proteins, and the PTP-C2 unit is homologous to the tumour suppressor PTEN [160,161]. The PTP domain of PTEN possesses an intact signature motif, and it displays phosphatase activity for both phospholipids and phosphotyrosine [162]. It is therefore possible that the PTP-like domain of tensin1 and auxilin may be a phosphotyrosine or phospholipid binding domain. Many PTP-like domains are awaiting functional characterization. 

#### Raf-1 kinase inhibitory protein

Raf-1 kinase inhibitory protein (RKIP), also known as phosphatidylethanolamine binding protein 1, suppresses Raf-1 kinase activity in the MAP kinase pathway [163]. RKIP binds to a 24 amino acid stretch in the N-terminal of the Raf-1 kinase which has a central element (S^338^SYY^341^) regulated by phosphorylation [164]. Substitutions in the ligand binding site of RKIP compromise the stability of the phosphorylated Raf-RKIP complex. Additionally, mutation of the Ser residues or Tyr341 on the Raf-1 kinase motif disrupts its interaction with RKIP. This suggests that RKIP contains a novel phospho-amino acid binding domain [165]. The structure of RKIP bound to pTyr shows that RKIP indeed contains a deep pocket molded complementary to the shape of the pTyr side chain (Figure 6B) [149]. NMR titration studies further confirmed that the pocket region is the binding site for the tri-phosphorylated Raf-1 peptide, pS^338^pSYpY, with a *K*_D_ value of 45 μM [166]. However, direct, physiological or structural, evidence for complex formation is required before RKIP can be classified as a *bona fide* pTyr binder.

#### Searching for novel pTyr recognition proteins

In the above sections, we have explored that, in addition to the SH2 and PTB domains, there are more than a handful of examples of atypical domains that can recognize and bind pTyr-containing target proteins. So does nature have more domain members capable of pTyr-epitope binding that remain to be identified? Recent advances in proteomic technologies are starting to shed light on this question. Christofk et al. employed the SILAC (stable isotope labeling of amino acids in cell culture) technique in combination with a pTyr-peptide library to identify novel pTyr-binding proteins from mammalian cell lysates [150]. Whereas the majority of proteins identified were SH2 or PTB domain-containing proteins, as expected, the screening also identified pyruvate kinase, a protein that does not have any known pTyr-recognition domain. Pyruvate kinase (PK) regulates the final rate-limiting step in glycolysis that converts cellular metabolism from an anaerobic to an aerobic process. The M2 isoform of the PK (PKM2) is the only form of PK used for glycolysis in cancer cells, and it is also the only one of the four isoforms that has the ability to bind to pTyr targets [167,168]. The structure of hPKM2 illustrates a homo-tetramer with each monomer binding a fructose-1,6-bisphosphate (FBP) molecule in a site distal to the active site (Figure 6C) [150,169]. Phosphotyrosine ligand binding may be a key event in modulating PK activity regulated by the allosteric activator FBP [150]. The study demonstrated that PKM2 binds pTyr peptides on the lip of the FBP binding pocket and acts as a negative regulator of PK activity (Figure 6C) [150]. Binding of the phosphopeptide releases FBP from the active tetrameric form of PKM2 and dissociates the enzyme into inactive dimers [168].

More recently, Christofk et al. identified a handful of potential pTyr-binding proteins from SILAC experiments. These include the serine/threonine-protein kinase WNK1, 5-formyltetrahydrofolate cyclo-ligase, glycerol-3-phosphate dehydrogenase, vimentin, 2,4-dienoyl-CoA reductase, and the T-complex protein 1 subunit η [170]. Although biochemical characterization is required to ascertain if these candidates are true pTyr-binders, results from this study suggest that there may be more pTyr recognition domains or proteins in nature that await to be discovered.

### Phosphotyrosine recognition domains and therapeutic applications

New insights into the molecular basis of cancer were generated already decades ago by the analysis of SH2-domain containing oncoproteins such as v-Src [27,171]. SH2-domain containing proteins have been implicated in many diseases, including immune-related disorders, metabolic syndromes, osteopathologic conditions and different cancers [10,172]. Mutations in the SH2 domains that cause malignancies fall usually into one of three categories: missense mutations of amino acids involved in target binding, mutations in residues that regulate catalytic activity or mutations on the SH2 domain, distal to the module core, that affect the architectural integrity of the SH2 domain [16]. A number of disease-causing mutations on SH2 domains have been reported as compiled by Liu et al. [10] and Lappalainen, et al. [173], including mutations related to the Noonan syndrome, juvenile myelomonocytic leukemia and the X-linked lymphoproliferative syndrome. X-chromosome-linked agammaglobulinemia (XLA) is an example of a disease where mutations are present in the SH2 domain of the Bruton's tyrosine kinase (BTK), and many of these mutations are located on the pTyr-ligand binding site, including the indispensable arginine of the pTyr-binding pocket as well as BG loop residues [16,173]. Moreover, Hong et al. demonstrated that the BTK SH2 domain binds to phospholipids and the XLA-causing mutations alter lipid binding selectivity [174]. In another example, multiple point mutations within the transcription factor STAT3 SH2 domain have been identified and linked to large granular lymphocytic leukemia and the hyper-IgE syndrome [175,176]. In conjunction with the identification and study of SH2 domain-related diseases, small molecule inhibitors of SH2 domains, SH2 domain-containing proteins or SH2 binding partners, are being developed with some success as therapeutic reagents, although the development of phosphomimetics faces hurdles due to the strong charge of the phosphate group [177,178]. Besides SH2 domains, mutations in the PTB domain have also been linked to diseases such as coronary heart disease and type II diabetes [85].

Biochemical and structural studies have demonstrated that direct intramolecular interactions between the SH2 and kinase domains are required for kinase activation in some PTKs [15,16,36]. In the Fes kinase, electrostatic interactions and shape complementarity between residues from the SH2 domain and the αC helix of the kinase domain stabilize the active state of the kinase. Similarly, in the Abl kinase, interaction between Ile164 of the SH2 domain and Thr291/Tyr331 in the kinase domain N lobe is essential for activity [179]. Subsequently, this interface has been investigated intensively as a target for cancer intervention. A successful approach was the creation of a protein-based agent called the monobody, derived from the fibronectin type III (FN3) domain with engineered loops designed for high affinity binding to a specific target molecule [180]. Koide and colleagues created a monobody engineered to selectively bind the SH2 domain of the Abl kinase [181]. They further generated another monobody that targets the SH2-kinase interface that involves Ile164 and then connected the two monobodies with a linker. The resulting tandem monobody disrupted the interface between the SH2 and kinase domains to inhibit the catalytic activity of the deregulated fusion kinase Bcr-Abl, both *in vitro* and *in vivo*[179].

Due to its independently folding nature, a modular domain can often be successfully used as a tool in proteomic research. Jadwin et al. termed this "domainomics” [182]. In particular, applications of phosphotyrosine-recognition domains have been reported for therapeutic and diagnostic purposes. SH2 and PTB domain-based probes have been developed and used to profile global pTyr landscapes using innovative assays such as the peptide dot blotting, Far Western blotting and oligonucleotide tagged multiplex (OTM) assays [183-186]. The OTM assay is designed for quantitative multiplexed profiling of tyrosine-phosphorylated proteins from cell extracts using DNA-tagged SH2 domains, and has been successfully harnessed to discriminate tyrosine phosphorylation states in tumour cell lines and leukemia samples from patients [184]. Similarly, Machida et al. reported application of domain-based probe technology to profile lung cancer cell lines, and demonstrated significant correlation between EGFR mutations and the Grb2 SH2 and ShcA PTB domain probe signals, suggesting a diagnostic value [187]. A potential for application of SH2 domains as non-invasive intracellular imaging or as inhibitor reagents has also been demonstrated. An SH2 domain from Grb2 or PLCγ1 fused with a protein transduction tag (the TAT tag) [188] was used for SH2 domain delivery into cells. These TAT-tagged SH2 domains showed anti-tumour effects [189,190]. In addition, the TAT-tagged Grb2 SH2 domain can be used as a molecular probe for monitoring EGFR localization in cells [191].

SH2 domains have also been incorporated as a biosensor molecule that functions in live cells. Wang et al. created a bipartite Src reporter biosensor protein that contains an SH2 domain and a Src kinase substrate fused, respectively, with the cyan and green fluorescent proteins [192]. Upon phosphorylation of the substrate by endogenous Src kinase, the SH2 domain of the biosensor binds to the substrate in *cis*, and produces a change in the emission spectrum. Using the Src reporter, the authors successfully visualized kinase activity in cells. In another example, an engineered adenocarcinoma-derived cell line that expresses an EGFR biosensor has been developed [193]. This cell line expresses the Grb2 SH2 domain fused to the green fluorescent protein, and can be used to monitor EGFR internalization upon EGF stimulation. The cell line may provide a useful tool for high-throughput drug screening since effects of drug candidates on modulating EGFR activity can be monitored in live cells.

**Figure 8:**
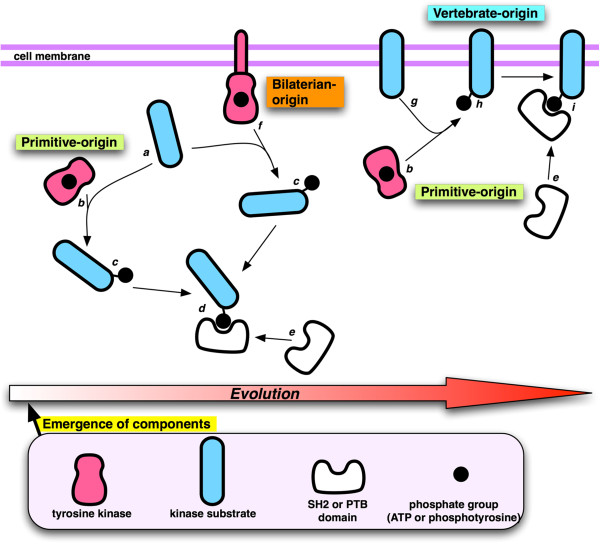
***Evolution of the human PTK signalling circuit.*** A schematic depicting the evolution of the human PTK circuit, where *a*, *b*, *c*, *d*, and *e* denote intracellular human circuits of a primitive origin, *a*, *f*, *c*, *d*, and *e* denote circuits with receptor tyrosine kinases and cytoplasmic substrates of bilaterian origin, and *g*, *b*, *h*, *i* and *e* denote vertebrate-origin circuits in which membrane-bound substrates are phosphorylated by cytoplasmic tyrosine kinases of a primitive origin in a tissue-specific manner. See reference [8] for more details.

Lastly, understanding the evolution of phosphotyrosine recognition domains may inform cancer research and treatment. In general, the degree of network complexity in the phosphotyrosine signaling system has increased during the course of evolution, and genetic events, such as gene duplications to create paralog proteins and gene fusions to create multi-domain proteins, played a major role in expanding the network scale [4,11]. Robustness of the signaling network is partly conferred by network redundancy and a collection of feedback loops or cross-talks [194]. The network evolution creates highly connected conserved nodes, called network hubs, and cancer-causing mutations tend to be observed for the network hub proteins [194]. Besides, Kitano argues that cancer cells hijack the cellular signaling mechanisms for their network robustness [195]. We have recently analyzed the evolutionary origins of human pTyr signalling circuits in 19 eukaryotic species by identifying ortholog proteins of the human circuit components in each species (Figure 8) [8]. The 19 species were classified into three groups (primitive organisms, bilaterians, and vertebrates) based on their evolutionary distances from humans. In this study, a pTyr signalling circuit is defined to comprise a tyrosine kinase, a substrate of the tyrosine kinase, and an SH2 or PTB domain that binds to the tyrosine-phosphorylated site. Statistical analysis showed that the circuits for intracellular (cytoplasmic) signalling (Figure 8: *a*, *b*, *c*, *d*, and *e*) largely originate from primitive species. Circuits that involve receptor tyrosine kinases which phosphorylate cytoplasmic substrates (Figure 8: *a*, *f*, *c*, *d*, and *e*) mainly originate from bilaterians. Conversely, vertebrate-origin circuits are enriched with membrane protein substrates that are phosphorylated by primitive-origin cytoplasmic kinases (Figure 8; *g*, *b*, *h*, *i* and *e*) in a tissue-specific manner. This study underlined the importance of network hubs as hotspots for tumourigenesis, as high frequency cancer pTyr sites are involved in more circuits than low frequency sites, and kinase substrate proteins that contain a PTK, SH2 or PTB domain are more frequently recruited for cancer signalling [8]. 

### Future perspectives

The genome sequence analysis of *M*. *brevicollis*, a unicellular choanoflagellate that possesses a PTK signalling system [70], brought a big surprise since the numbers of PTKs, PTPs and SH2 domains are comparable to or greater than those in mammals [6,100]. Numerous PTK, PTP, SH2 and PTB domain-containing proteins have been also identified in other pre-metazoan species [196,197], challenging the simple notion of "expansion from yeast to man" in terms of the evolution of phosphotyrosine signalling. Since *M*. *brevicollis* contains a plethora of putative PTK toolkit proteins, many of which have no orthologs in humans [6,100,196], it suggests that multiple evolutionary roads, which remain to be elucidated, may have been used to mix and match different pTyr signalling components in order to build the PTK signalling pathways and networks unique to a particular species. For example, bacteria may possess their own phosphotyrosine signalling systems as bacterial tyrosine kinases are found to be unrelated to eukaryotic PTKs [198,199]. Nevertheless, bacteria do not only rely on their own repertoire of pTyr signalling components. Some pathogenic bacteria, for example the enteropathongenic *Escherichia coli* (EPEC) and *Helicobacter pylori*, are known to hijack pTyr signalling system components of their host by injecting a virulence factor into the host cell during infection [200,201]. The injected bacterial protein is then phosphorylated by a host PTK, and subsequently recruits SH2 domains of host proteins to rewire the host signalling network for the bacteria's benefit. The injected bacterial effectors have evolved to be readily phosphorylated by host PTKs, and to recruit a number of host SH2 domains after being phosphorylated [201,202]. For instance, EPEC injects a transmembrane effector protein Tir (translocated intimin receptor) to host cells upon infection. The cytoplasmic YDXV motif of Tir is then phosphorylated and recruits the SH2 domain of the adaptor protein NCK, which results in pathological actin reorganization [200,203]. It is noteworthy that a similar scenario is seen in the vaccinia virus. A tyrosine residue in the viral membrane protein named A36R is phosphorylated by host PTKs, and the viral protein subsequently recruits the NCK and Grb2 SH2 domains to manipulate downstream actin regulation of the host cell [204,205]. Intervention of pTyr-mediated host-pathogen interactions could be a novel therapeutic strategy, if sufficiently selective agents can be developed.

## Conclusions

Much of what has been learnt in past decades about pTyr reader domains, their interactions and the networks that they serve can now be utilised and also warrants further exploration into several directions. Firstly, we need to make an effort to put more ‘puzzle pieces’ together to start seeing a more complete picture. This includes analysing in much more detail how the compartmentalisation of pTyr writers and readers, i.e. the architecture of the pTyr signalling machinery, generates highly specific signals *in vivo*. Protein modification reader domains should also be useful in a range of clinical settings, for example for molecular diagnoses and monitoring, but this potential remains untapped in clinical routine settings. As demonstrated for the SH2 domains, e.g., specificity-switching mutants or affinity-enhanced "pTyr superbinders" [40,61,63], engineering of pTyr-binding domains may yield protein-based reagents with desired specificities and affinities towards tyrosine phosphorylated targets, which may provide an array of novel agents for research and clinical purposes. Furthermore, we are just beginning to realise that at least some protein modification reader domains have the capacity to decipher combinations of modifications rather than recognising just single site changes, and further research in this area promises to provide additional clues to understanding the elegant ways in which cells manage to read and process a huge number of incoming signals and to translate them into a wide spectrum of biochemical and biological responses. Hence, typical and atypical pTyr reader domains should be considered to be ‘old dogs up to new tricks’ which we are just starting to grasp.

## Competing interests

The authors declare that they have no competing interests.

## Authors’ contributions

RJ and TK drafted the first version of the manuscript. All authors edited and approved the final version.

## References

[B1] Seet BT Dikic I Zhou MM Pawson T Reading protein modifications with interaction domains Nat Rev Mol Cell Biol 2006 7 473 483 16829979 10.1038/nrm1960

[B2] Lahiry P Torkamani A Schork NJ Hegele RA Kinase mutations in human disease: interpreting genotype-phenotype relationships Nat Rev Genet 2010 11 60 74 10.1038/nrg2707 20019687

[B3] Hunter T Tyrosine phosphorylation: thirty years and counting Curr Opin Cell Biol 2009 21 140 146 10.1016/j.ceb.2009.01.028 19269802 PMC2670436

[B4] Jin J Xie X Chen C Park JG Stark C James DA Olhovsky M Linding R Mao Y Pawson T Eukaryotic protein domains as functional units of cellular evolution Sci Signal 2009 2 ra76 10.1126/scisignal.2000546 19934434

[B5] Pawson T Nash P Assembly of cell regulatory systems through protein interaction domains Science 2003 300 445 452 10.1126/science.1083653 12702867

[B6] Pincus D Ivica L Peer B Lim WA Evolution of the phospho-tyrosine signaling machinery in premetazoan lineages Proc Natl Acad Sci U S A 2008 105 9680 9684 10.1073/pnas.0803161105 18599463 PMC2443182

[B7] Lim WA Pawson T Phosphotyrosine signaling: evolving a new cellular communication system Cell 2010 142 661 667 10.1016/j.cell.2010.08.023 20813250 PMC2950826

[B8] Li L Tibiche C Fu C Kaneko T Moran MF Schiller MR Li SS Wang E The human phosphotyrosine signalling network: Evolution and hotspots of hijacking in cancer Genome Res 2012 22 1222 1230 10.1101/gr.128819.111 22194470 PMC3396364

[B9] Manning G Whyte DB Martinez R Hunter T Sudarsanam S The protein kinase complement of the human genome Science 2002 298 1912 1934 10.1126/science.1075762 12471243

[B10] Liu BA Jablonowski K Raina M Arce M Pawson T Nash PD The human and mouse complement of SH2 domain proteins-establishing the boundaries of phosphotyrosine signalling Mol Cell 2006 22 851 868 10.1016/j.molcel.2006.06.001 16793553

[B11] Liu BA Shah E Jablonowski K Stergachis A Engelmann B Nash PD The SH2 domain-containing proteins in 21 species establish the provenance and scope of phosphotyrosine signalling in eukaryotes Sci Signal 2011 4 ra83 10.1126/scisignal.2002105 22155787 PMC4255630

[B12] Liu BA Engelmann BW Nash PD The Language of SH2 Domain Interactions Defines Phosphotyrosine-Mediated Signal Transduction FEBS Lett 2012 586 2597 10.1016/j.febslet.2012.04.054 22569091

[B13] Tan CS Bodenmiller B Pasculescu A Jovanovic M Hengartner MO Jorgensen C Bader GD Aebersold R Pawson T Linding R Comparative analysis reveals conserved protein phosphorylation networks implicated in multiple diseases Sci Signal 2009 2 ra39 10.1126/scisignal.2000316 19638616

[B14] Lemmon MA Schlessinger J Cell Signaling by Receptor Tyrosine Kinases Cell 2010 141 1117 1134 10.1016/j.cell.2010.06.011 20602996 PMC2914105

[B15] Pawson T Kofler M Kinome signalling through regulated protein-protein interactions in normal and cancer cells Curr Opin Cell Biol 2009 21 147 153 10.1016/j.ceb.2009.02.005 19299117

[B16] Filippakopoulos P Muller S Knapp S SH2 domains: modulators of nonreceptor tyrosine kinase activity Curr Opin Struct Biol 2009 19 643 649 10.1016/j.sbi.2009.10.001 19926274 PMC2791838

[B17] Mayer BJ Perspective: Dynamics of receptor tyrosine kinase signalling complexes FEBS Lett 2012 586 2575 2579 10.1016/j.febslet.2012.05.002 22584051 PMC3413745

[B18] Songyang Z Carraway IKL Eck MJ Catalytic specificity of protein- tyrosine kinase is critical for selective signalling Nature 1995 373 536 539 10.1038/373536a0 7845468

[B19] Songyang Z Cantley LC Recognition and specificity in protein tyrosine kinase-mediated signalling Trends Biochem Sci 1995 20 470 475 10.1016/S0968-0004(00)89103-3 8578591

[B20] Huang H Li L Wu C Schibli D Colwill K Ma S Li C Roy P Ho K Songyang Z Defining the specificity space of the human SRC homology 2 domain Mol Cell Proteomics 2008 7 768 784 17956856 10.1074/mcp.M700312-MCP200

[B21] Miller ML Jensen LJ Diella F Jorgensen C Tinti M Li L Hsiung M Parker SA Bordeaux J Sicheritz-Ponten T Linear motif atlas for phosphorylation-dependent signalling Sci Signal 2008 1 ra2 10.1126/scisignal.1159433 18765831 PMC6215708

[B22] Wavreille AS Garaud M Zhang Y Pei D Defining SH2 domain and PTP specificity by screening combinatorial peptide libraries Methods 2007 42 207 219 10.1016/j.ymeth.2007.02.010 17532507 PMC2041848

[B23] Wolf-Yadlin A Sevecka M MacBeath G Dissecting protein function and signalling using protein microarrays Curr Opin Chem Biol 2009 13 398 405 10.1016/j.cbpa.2009.06.027 19660979 PMC2811369

[B24] Scott JD Pawson T Cell signalling in space and time: where proteins come together and when they're apart Science 2009 326 1220 1224 10.1126/science.1175668 19965465 PMC3041271

[B25] Bhattacharya R Domains, Motifs, and Scaffolds: The Role of Modular Interactions in the Evolution and Wiring of Cell Signaling Circuits Annu Rev Biochem 2006 75 655 680 10.1146/annurev.biochem.75.103004.142710 16756506

[B26] Sadowski I Stone JC Pawson T A noncatalytic domain conserved among cytoplasmic protein-tyrosine kinases modifies the kinase function and transforming activity of Fujinami sarcoma virus P130gag-fps Mol Cell Biol 1986 6 4396 4408 3025655 10.1128/mcb.6.12.4396-4408.1986 PMC367222

[B27] Pawson T Specificity in Signal Transduction: From Phosphotyrosine-SH2 Domain Interactions to Complex Cellular Systems Cell 2004 116 191 203 10.1016/S0092-8674(03)01077-8 14744431

[B28] Mayer BJ Hamaguchi M Hanafusa H A novel viral oncogene with structural similarity to phospholipase C Nature 1988 332 272 275 10.1038/332272a0 2450282

[B29] Anderson D Koch CA Grey L Ellis C Moran MF Pawson T Binding of SH2 domains of phospholipase C gamma 1, GAP, and Src to activated growth factor receptors Science 1990 250 979 982 10.1126/science.2173144 2173144

[B30] Pawson T Gish GD SH2 and SH3 domains: from structure to function Cell 1992 71 359 362 10.1016/0092-8674(92)90504-6 1423600

[B31] Matsuda M Mayer BJ Fukui Y Hanafusa H Binding of transforming protein, P47gag-crk, to a broad range of phosphotyrosine-containing proteins Science 1990 248 1537 1539 10.1126/science.1694307 1694307

[B32] Matsuda M Mayer BJ Hanafusa H Identification of domains of the v-crk oncogene product sufficient for association with phosphotyrosine-containing proteins Mol Cell Biol 1991 11 1607 1613 1705010 10.1128/mcb.11.3.1607-1613.1991 PMC369454

[B33] Mayer BJ Jackson PK Baltimore D The noncatalytic src homology region 2 segment of abl tyrosine kinase binds to tyrosine-phosphorylated cellular proteins with high affinity Proc Natl Acad Sci U S A 1991 88 627 631 10.1073/pnas.88.2.627 1703304 PMC50865

[B34] Muller AJ Pendergast AM Havlik MH Puil L Pawson T Witte ON A limited set of SH2 domains binds BCR through a high-affinity phosphotyrosine-independent interaction Mol Cell Biol 1992 12 5087 5093 1383690 10.1128/mcb.12.11.5087-5093.1992 PMC360442

[B35] Moran MF Koch CA Anderson D Ellis C England L Martin GS Pawson T Src homology region 2 domains direct protein-protein interactions in signal transduction Proc Natl Acad Sci U S A 1990 87 8622 8626 10.1073/pnas.87.21.8622 2236073 PMC55009

[B36] Filippakopoulos P Kofler M Hantschel O Gish GD Grebien F Salah E Neudecker P Kay LE Turk BE Superti-Furga G Structural coupling of SH2-kinase domains links Fes and Abl substrate recognition and kinase activation Cell 2008 134 793 803 10.1016/j.cell.2008.07.047 18775312 PMC2572732

[B37] Heldin CH SH2 domains: elements that control protein interactions during signal transduction Trends Biochem Sci 1991 16 450 452 1781020 10.1016/0968-0004(91)90175-u

[B38] Waksman G Kominos D Robertson SC Pant N Baltimore D Birge RB Cowburn D Hanafusa H Mayer BJ Overduin M Crystal structure of the phosphotyrosine recognition domain SH2 of v-src complexed with tyrosine-phosphorylated peptides Nature 1992 358 646 653 10.1038/358646a0 1379696

[B39] Waksman G Shoelson SE Pant N Cowburn D Kuriyan J Binding of a high affinity phosphotyrosyl peptide to the Src SH2 domain: crystal structures of the complexed and peptide-free forms Cell 1993 72 779 790 10.1016/0092-8674(93)90405-F 7680960

[B40] Kaneko T Huang H Zhao B Li L Liu H Voss CK Wu C Schiller MR Li SS Loops govern SH2 domain specificity by controlling access to binding pockets Sci Signal 2010 3 ra34 10.1126/scisignal.2000796 20442417 PMC6590088

[B41] Waksman G Kuriyan J Structure and specificity of the SH2 domain Cell 2004 S116 S45 S48 15055581 10.1016/s0092-8674(04)00043-1

[B42] Feller SM Ren R Hanafusa H Baltimore D SH2 and SH3 domains as molecular adhesives: the interactions of Crk and Abl Trends Biochem Sci 1994 19 453 458 10.1016/0968-0004(94)90129-5 7855886

[B43] Meng W Structure of the amino-terminal domain of Cbl complexed to its binding site on ZAP-70 kinase Nature 1999 398 84 90 10.1038/18050 10078535

[B44] Mallis RJ Brazin KN Fulton DB Andreotti AH Structural characterization of a proline-driven conformational switch within the Itk SH2 domain Nat Struct Biol 2002 9 900 905 10.1038/nsb864 12402030

[B45] Severin A Joseph RE Boyken S Fulton DB Andreotti AH Proline isomerization preorganizes the Itk SH2 domain for binding to the Itk SH3 domain J Mol Biol 2009 387 726 743 10.1016/j.jmb.2009.02.012 19361414 PMC2810249

[B46] Joseph RE Ginder ND Hoy JA Nix JC Fulton DB Honzatko RB Andreotti AH Structure of the interleukin-2 tyrosine kinase Src homology 2 domain; comparison between X-ray and NMR-derived structures Acta crystallographica Section F 2012 68 145 153 10.1107/S1744309111049761 PMC3274390 22297986

[B47] Donaldson LW Gish G Pawson T Kay LE Forman-Kay JD Structure of a regulatory complex involving the Abl SH3 domain, the Crk SH2 domain, and a Crk-derived phosphopeptide Proc Natl Acad Sci U S A 2002 99 14053 14058 10.1073/pnas.212518799 12384576 PMC137835

[B48] Close D Johnson SJ Sdano MA McDonald SM Robinson H Formosa T Hill CP Crystal structures of the S. cerevisiae Spt6 core and C-terminal tandem SH2 domain J Mol Biol 2011 408 697 713 10.1016/j.jmb.2011.03.002 21419780 PMC3086336

[B49] Sun M Lariviere L Dengl S Mayer A Cramer P A tandem SH2 domain in transcription elongation factor Spt6 binds the phosphorylated RNA polymerase II C-terminal repeat domain (CTD) J Biol Chem 2010 285 41597 41603 10.1074/jbc.M110.144568 20926372 PMC3009887

[B50] Liu J Zhang J Gong Q Xiong P Huang H Wu B Lu G Wu J Shi Y Solution structure of tandem SH2 domains from Spt6 protein and their binding to the phosphorylated RNA polymerase II C-terminal domain J Biol Chem 2011 286 29218 29226 10.1074/jbc.M111.252130 21676864 PMC3190728

[B51] Diebold ML Loeliger E Koch M Winston F Cavarelli J Romier C Noncanonical tandem SH2 enables interaction of elongation factor Spt6 with RNA polymerase II J Biol Chem 2010 285 38389 38398 10.1074/jbc.M110.146696 20926373 PMC2992272

[B52] Yoh SM Cho H Pickle L Evans RM Jones KA The Spt6 SH2 domain binds Ser2-P RNAPII to direct Iws1-dependent mRNA splicing and export Genes Dev 2007 21 160 174 10.1101/gad.1503107 17234882 PMC1770899

[B53] Songyang Z Shoelson SE Chaudhuri M Gish G Pawson T Haser WG King F Roberts T Ratnofsky S Lechleider RJ SH2 domains recognize specific phosphopeptide sequences Cell 1993 72 767 778 10.1016/0092-8674(93)90404-E 7680959

[B54] Songyang Z Shoelson SE McGlade J Olivier P Pawson T Bustelo XR Barbacid M Sabe H Hanafusa H Yi T Specific motifs recognized by the SH2 domains of Csk, 3BP2, fps/fes, GRB-2, HCP, SHC, Syk, and Vav Mol Cell Biol 1994 14 2777 2785 10.1128/MCB.14.4.2777 7511210 PMC358643

[B55] Songyang Z Cantley LC ZIP codes for delivering SH2 domains Cell 2004 S116 S41 S43 15055580 10.1016/s0092-8674(04)00041-8

[B56] Ladbury JE Arold ST Energetics of Src homology domain interactions in receptor tyrosine kinase-mediated signalling Methods Enzymol 2011 488 147 183 21195228 10.1016/B978-0-12-381268-1.00007-0

[B57] Jones RB Gordus A Krall JA MacBeath G A quantitative protein interaction network for the ErbB receptors using protein microarrays Nature 2006 439 168 174 10.1038/nature04177 16273093

[B58] Kaushansky A Gordus A Chang B Rush J MacBeath G A quantitative study of the recruitment potential of all intracellular tyrosine residues on EGFR, FGFR1 and IGF1R Mol Biosyst 2008 4 643 653 10.1039/b801018h 18493663 PMC2811368

[B59] Kaushansky A Gordus A Budnik BA Lane WS Rush J MacBeath G System-wide investigation of ErbB4 reveals 19 sites of Tyr phosphorylation that are unusually selective in their recruitment properties Chem Biol 2008 15 808 817 10.1016/j.chembiol.2008.07.006 18721752 PMC2606095

[B60] Hause RJ Jr Leung KK Barkinge JL Ciaccio MF Chuu CP Jones RB Comprehensive Binary Interaction Mapping of SH2 Domains via Fluorescence Polarization Reveals Novel Functional Diversification of ErbB Receptors PLoS One 2012 7 e44471 10.1371/journal.pone.0044471 22973453 PMC3433420

[B61] Kaneko T Huang H Cao X Li X Li C Voss C Sidhu SS Li SS Superbinder SH2 Domains Act as Antagonists of Cell Signaling Sci Signal 2012 5 ra68 10.1126/scisignal.2003021 23012655

[B62] Waksman G Kumaran S Lubman O SH2 domains: role, structure and implications for molecular medicine Expert Rev Mol Med 2004 6 1 18 14987415 10.1017/S1462399404007331

[B63] Marengere LE Songyang Z Gish GD Schaller MD Parsons JT Stern MJ Cantley LC Pawson T SH2 domain specificity and activity modified by a single residue Nature 1994 369 502 505 10.1038/369502a0 7515480

[B64] Kaneko T Sidhu SS Li SS Evolving specificity from variability for protein interaction domains Trends Biochem Sci 2011 36 183 190 10.1016/j.tibs.2010.12.001 21227701

[B65] Liu BA Jablonowski K Shah EE Engelmann BW Jones RB Nash PD SH2 domains recognize contextual peptide sequence information to determine selectivity Mol Cell Proteomics 2010 9 2391 2404 10.1074/mcp.M110.001586 20627867 PMC2984226

[B66] Rodriguez M Li SS Harper JW Songyang Z An oriented peptide array library (OPAL) strategy to study protein-protein interactions J Biol Chem 2004 279 8802 8807 10.1074/jbc.M311886200 14679191

[B67] Egloff S Murphy S Cracking the RNA polymerase II CTD code Trends Genet 2008 24 280 288 10.1016/j.tig.2008.03.008 18457900

[B68] Hsin JP Sheth A Manley JL RNAP II CTD phosphorylated on threonine-4 is required for histone mRNA 3' end processing Science 2011 334 683 686 10.1126/science.1206034 22053051 PMC3678764

[B69] Dengl S Mayer A Sun M Cramer P Structure and in vivo requirement of the yeast Spt6 SH2 domain J Mol Biol 2009 389 211 225 10.1016/j.jmb.2009.04.016 19371747

[B70] King N Hittinger CT Carroll SB Evolution of key cell signalling and adhesion protein families predates animal origins Science 2003 301 361 363 10.1126/science.1083853 12869759

[B71] Raffel GD Parmar K Rosenberg N In vivo association of v-Abl with Shc mediated by a non-phosphotyrosine-dependent SH2 interaction J Biol Chem 1996 271 4640 4645 10.1074/jbc.271.9.4640 8617726

[B72] Dutartre H Harris M Olive D Collette Y The human immunodeficiency virus type 1 Nef protein binds the Src-related tyrosine kinase Lck SH2 domain through a novel phosphotyrosine independent mechanism Virology 1998 247 200 211 10.1006/viro.1998.9244 9705913

[B73] Hwang PM Li C Morra M Lillywhite J Muhandiram DR Gertler F Terhorst C Kay LE Pawson T Forman-Kay JD Li S-C A "three-pronged" binding mechanism for the SAP/SH2D1A SH2 domain: structural basis and relevance to the XLP syndrome EMBO J 2002 21 314 323 10.1093/emboj/21.3.314 11823424 PMC125837

[B74] Liao Y-C Si L deVere White RW Lo SH The phosphotyrosine-independent interaction of DLC-1 and the SH2 domain of cten regulates focal adhesion localization and growth suppression activity of DLC-1 J Cell Biol 2007 176 43 49 10.1083/jcb.200608015 17190795 PMC2063623

[B75] Qian X Li G Asmussen HK Asnaghi L Vass WC Braverman R Yamada KM Popescu NC Papageorge AG Lowy DR Oncogenic inhibition by a deleted in liver cancer gene requires cooperation between tensin binding and Rho-specific GTPase-activating protein activities Proc Natl Acad Sci U S A 2007 104 9012 9017 10.1073/pnas.0703033104 17517630 PMC1868654

[B76] Bae JH Lew ED Yuzawa S Tome F Lax I Schlessinger J The selectivity of receptor tyrosine kinase signalling is controlled by a secondary SH2 domain binding site Cell 2009 138 514 524 10.1016/j.cell.2009.05.028 19665973 PMC4764080

[B77] Min L Joseph RE Fulton DB Andreotti AH Itk tyrosine kinase substrate docking is mediated by a nonclassical SH2 domain surface of PLCgamma1 Proc Natl Acad Sci U S A 2009 106 21143 21148 10.1073/pnas.0911309106 19955438 PMC2786894

[B78] Dai K Liao S Zhang J Zhang X Tu X Solution structure of tensin2 SH2 domain and its phosphotyrosine-independent interaction with DLC-1 PLoS One 2011 6 e21965 10.1371/journal.pone.0021965 21765928 PMC3134462

[B79] Poy F Crystal Structures of the XLP Protein SAP Reveal a Class of SH2 Domains with Extended, Phosphotyrosine-Independent Sequence Recognition Mol Cell 1999 4 555 561 10.1016/S1097-2765(00)80206-3 10549287

[B80] Fang Y Johnson LM Mahon ES Anderson DH Two phosphorylation-independent sites on the p85 SH2 domains bind A-Raf kinase Biochem Biophys Res Commun 2002 290 1267 1274 10.1006/bbrc.2002.6347 11812000

[B81] Robinson DR The protein tyrosine kinase family of the human genome Oncogene 2000 19 5548 5557 10.1038/sj.onc.1203957 11114734

[B82] Mayer BJ Hirai H Sakai R Evidence that SH2 domains promote processive phosphorylation by protein-tyrosine kinases Current biology: CB 1995 5 296 305 10.1016/S0960-9822(95)00060-1 7780740

[B83] Colicelli J ABL tyrosine kinases: evolution of function, regulation, and specificity Sci Signal 2010 3 re6 10.1126/scisignal.3139re6 20841568 PMC2954126

[B84] Akiva E Friedlander G Itzhaki Z Margalit H A dynamic view of domain-motif interactions PLoS Comput Biol 2012 8 e1002341 10.1371/journal.pcbi.1002341 22253583 PMC3257277

[B85] Uhlik MT Temple B Bencharit S Kimple AJ Siderovski DP Johnson GL Structural and evolutionary division of phosphotyrosine binding (PTB) domains J Mol Biol 2005 345 1 20 10.1016/j.jmb.2004.10.038 15567406

[B86] Pei D Lorenz U Klingmuller U Neel BG Walsh CT Intramolecular regulation of protein tyrosine phosphatase SH-PTP1: a new function for Src homology 2 domains Biochemistry 1994 33 15483 15493 10.1021/bi00255a030 7528537

[B87] Hof P Pluskey S Crystal Structure of the Tyrosine Phosphatase SHP-2 Cell 1998 92 441 450 10.1016/S0092-8674(00)80938-1 9491886

[B88] Yang J Liu L He D Song X Liang X Zhao ZJ Zhou GW Crystal structure of human protein-tyrosine phosphatase SHP-1 J Biol Chem 2003 278 6516 6520 10.1074/jbc.M210430200 12482860

[B89] Wang W Liu L Song X Mo Y Komma C Bellamy HD Zhao ZJ Zhou GW Crystal structure of human protein tyrosine phosphatase SHP-1 in the open conformation J Cell Biochem 2011 112 2062 2071 10.1002/jcb.23125 21465528 PMC3135737

[B90] Zhang Y Jinjin Z Chunhua Y Ryan LH In-Hee P Chenglong L Charles B Pei D Simultaneous Binding of Two Peptidyl Ligands by a Src Homology 2 Domain Biochemistry 2011 50 7637 7646 10.1021/bi200439v 21800896 PMC3164524

[B91] Kesti T Ruppelt A Wang J-H Liss M Wagner R Taskén K Saksela K Reciprocal regulation of SH3 and SH2 domain binding via tyrosine phosphorylation of a common site in CD3epsilon J Immunol 2007 179 878 885 17617578 10.4049/jimmunol.179.2.878

[B92] Aitio O Hellman M Kesti T Kleino I Samuilova O Pääkkönen K Tossavainen H Saksela K Permi P Structural basis of PxxDY motif recognition in SH3 binding J Mol Biol 2008 382 167 178 10.1016/j.jmb.2008.07.008 18644376

[B93] Takeuchi K Yang H Ng E Park S-y Sun Z-YJ Reinherz EL Wagner G Structural and functional evidence that Nck interaction with CD3epsilon regulates T-cell receptor activity J Mol Biol 2008 380 704 716 10.1016/j.jmb.2008.05.037 18555270 PMC2577852

[B94] Wang Z Sandiford S Wu C Li SS Numb regulates cell-cell adhesion and polarity in response to tyrosine kinase signalling EMBO J 2009 28 2360 2373 10.1038/emboj.2009.190 19609305 PMC2712596

[B95] Fujita Y Krause G Scheffner M Zechner D Leddy HE Behrens J Sommer T Birchmeier W Hakai, a c-Cbl-like protein, ubiquitinates and induces endocytosis of the E-cadherin complex Nat Cell Biol 2002 4 222 231 10.1038/ncb758 11836526

[B96] Mukherjee M Chow SY Yusoff P Seetharaman J Ng C Sinniah S Koh XW Asgar NF Li D Yim D Structure of a novel phosphotyrosine-binding domain in Hakai that targets E-cadherin EMBO J 2012 31 1308 1319 10.1038/emboj.2011.496 22252131 PMC3298002

[B97] Blaikie P Immanuel D Wu J Li N Yajnik V Margolis B A region in Shc distinct from the SH2 domain can bind tyrosine-phosphorylated growth factor receptors J Biol Chem 1994 269 32031 32034 7798194

[B98] Kavanaugh WM Williams LT An alternative to SH2 domains for binding tyrosine-phosphorylated proteins Science 1994 266 1862 1865 10.1126/science.7527937 7527937

[B99] Forman-Kay JD Pawson T Diversity in protein recognition by PTB domains Curr Opin Struct Biol 1999 9 690 695 10607674 10.1016/s0959-440x(99)00031-7

[B100] Manning G Young SL Miller WT Zhai Y The protist, Monosiga brevicollis, has a tyrosine kinase signalling network more elaborate and diverse than found in any known metazoan Proc Natl Acad Sci U S A 2008 105 9674 9679 10.1073/pnas.0801314105 18621719 PMC2453073

[B101] Yaffe M Phosphotyrosine-Binding Domains in Signal Transduction Nat Rev Mol Cell Biol 2002 3 177 186 10.1038/nrm759 11994738

[B102] Prieto-Echague V Chan PM Craddock BP Manser E Miller WT PTB domain-directed substrate targeting in a tyrosine kinase from the unicellular choanoflagellate Monosiga brevicollis PLoS One 2011 6 e19296 10.1371/journal.pone.0019296 21541291 PMC3082566

[B103] Lemmon MA Membrane recognition by phospholipid-binding domains Nat Rev Mol Cell Biol 2008 9 99 111 10.1038/nrm2328 18216767

[B104] Li SC Zwahlen C Vincent SJ McGlade CJ Kay LE Pawson T Forman-Kay JD Structure of a Numb PTB domain-peptide complex suggests a basis for diverse binding specificity NatStruct Biol 1998 5 1075 1083 10.1038/4185 9846878

[B105] Zhou MM Huang B Olejniczak ET Meadows RP Shuker SB Miyazaki M Trub T Shoelson SE Fesik SW Structural basis for IL-4 receptor phosphopeptide recognition by the IRS-1 PTB domain Nat Struct Biol 1996 3 388 393 10.1038/nsb0496-388 8599766

[B106] Zhou MM Ravichandran KS Olejniczak EF Petros AM Meadows RP Sattler M Structure and ligand recognition of the phosphotyrosine binding domain of Shc Nature 1995 378 584 592 10.1038/378584a0 8524391

[B107] Dhe-Paganon Ottinger EA Nolte RT Eck MJ Shoelsen SE Crystal structure of the pleckstrin homology-phosphotyrosine binding (PH-PTB) targeting region of insulin receptor substrate 1 Proc Natl Acad Sci U S A 1999 96 8378 8383 10.1073/pnas.96.15.8378 10411883 PMC17524

[B108] Stolt PC Jeon H Song HK Herz J Eck MJ Blacklow SC Origins of Peptide Selectivity and Phosphoinositide Binding Revealed by Structures of Disabled-1 PTB Domain Complexes Structure 2003 11 569 579 10.1016/S0969-2126(03)00068-6 12737822

[B109] Yun M Keshvara L Park CG Zhang YM Dickerson JB Zheng J Rock CO Curran T Park HW Crystal structures of the Dab homology domains of mouse disabled 1 and 2 J Biol Chem 2003 278 36572 36581 10.1074/jbc.M304384200 12826668

[B110] DiNitto JP Lambright DG Membrane and juxtamembrane targeting by PH and PTB domains Biochim Biophys Acta 2006 1761 850 867 10.1016/j.bbalip.2006.04.008 16807090

[B111] Zwahlen C Li SC Kay LE Pawson T Forman-Kay JD Multiple modes of peptide recognition by the PTB domain of the cell fate determinant Numb EMBO J 2000 19 1505 1515 10.1093/emboj/19.7.1505 10747019 PMC310220

[B112] Chen L Liu C Ko FC Xu N Ng IO Yam JW Zhu G Solution structure of the PTB domain of human Tensin2 in complex with deleted in liver cancer 1 (DLC1) peptide reveals a novel peptide binding mode J Biol Chem 2012 287 26104 26114 10.1074/jbc.M112.360206 22645138 PMC3406694

[B113] Schulze WX Deng L Mann M Phosphotyrosine interactome of the ErbB-receptor kinase family Mol Syst Biol 2005 1 13 10.1038/msb4100012 PMC1681463 16729043

[B114] Smith MJ Hardy WR Murphy JM Jones N Pawson T Screening for PTB domain binding partners and ligand specificity using proteome-derived NPXY peptide arrays Mol Cell Biol 2006 26 8461 8474 10.1128/MCB.01491-06 16982700 PMC1636785

[B115] Rameh LE Arvidsson A-K Carraway IKL Couvillon AD Rathbun G Cromptoni A VanRenterghem B Czech MP Ravichandran KS Burakoff SJ A comparative analysis of the phosphoinositide binding specificity of pleckstrin homology domains J Biol Chem 1997 272 22059 22066 10.1074/jbc.272.35.22059 9268346

[B116] Guo M Jan LY Jan YN Control of daughter cell fates during asymmetric division:interaction of Numb and Notch Neuron 1996 17 27 41 10.1016/S0896-6273(00)80278-0 8755476

[B117] Qin H Percival-Smith A Li C Jia CY Gloor G Li SS A novel transmembrane protein recruits numb to the plasma membrane during asymmetric cell division J Biol Chem 2004 279 11304 11312 10.1074/jbc.M311733200 14670962

[B118] Nie J Li SS McGlade CJ A novel PTB-PDZ domain interaction mediates isoform-specific ubiquitylation of mammalian Numb J Biol Chem 2004 279 20807 20815 10.1074/jbc.M311396200 14990566

[B119] Wang Z Li SC Numb: A new player in EMT Cell Adh Migr 2010 4 176 179 10.4161/cam.4.2.10690 20168079 PMC2900608

[B120] Alonso A Sasin J Bottini N Friedberg I Friedberg I Osterman A Protein tyrosine phosphatases in the human genome Cell 2004 117 699 711 10.1016/j.cell.2004.05.018 15186772

[B121] Haynie DT Ponting CP The N-terminal domains of tensin and auxilin are phosphatase homologues Protein Sci 1996 5 2643 2646 10.1002/pro.5560051227 8976573 PMC2143309

[B122] Lo SH Tensin Int J Biochem Cell Biol 2004 36 31 34 10.1016/S1357-2725(03)00171-7 14592531

[B123] Song MS Salmena L Pandolfi PP The functions and regulation of the PTEN tumour suppressor Nat Rev Mol Cell Biol 2012 13 283 296 10.1038/nrg3199 22473468

[B124] Cao X Voss C Zhao B Kaneko T Li SSC Differential regulation of the activity of deleted in liver cancer 1 (DLC1) by tensins controls cell migration and transformation Proc Natl Acad Sci U S A 2012 109 1455 1460 10.1073/pnas.1114368109 22307599 PMC3277110

[B125] Qian X Li G Vass WC Papageorge A Walker RC Asnaghi L Steinbach PJ Tosato G Hunter K Lowy DR The Tensin-3 protein, including its SH2 domain, is phosphorylated by Src and contributes to tumorigenesis and metastasis Cancer Cell 2009 16 246 258 10.1016/j.ccr.2009.07.031 19732724 PMC3293497

[B126] McCleverty CJ Lin DC Liddington RC Structure of the PTB domain of tensin1 and a model for its recruitment to fibrillar adhesions Protein Sci 2007 16 1223 1229 10.1110/ps.072798707 17473008 PMC2206669

[B127] Calderwood DA Fujioka Y de Pereda JM Garcia-Alvarez B Nakamoto T Margolis B McGlade CJ Liddington RC Ginsberg MH Integrin beta cytoplasmic domain interactions with phosphotyrosine-binding domains: a structural prototype for diversity in integrin signalling Proc Natl Acad Sci U S A 2003 100 2272 2277 10.1073/pnas.262791999 12606711 PMC151330

[B128] Figueroa A Fujita Y Gorospe M Hacking RNA: Hakai promotes tumorigenesis by enhancing the RNA-binding function of PSF Cell Cycle 2009 8 3648 3651 10.4161/cc.8.22.9909 19855157 PMC2808762

[B129] Rodriguez-Rigueiro T Valladares-Ayerbes M Haz-Conde M Aparicio LA Figueroa A Hakai reduces cell-substratum adhesion and increases epithelial cell invasion BMC Cancer 2011 11 474 10.1186/1471-2407-11-474 22051109 PMC3229560

[B130] Katoh K Asimenos G Toh H Multiple alignment of DNA sequences with MAFFT Methods Mol Biol 2009 537 39 64 10.1007/978-1-59745-251-9_3 19378139

[B131] Mayer BJ Ren R Clark KL Baltimore D A putative modular domain present in diverse signalling molecules Cell 1993 73 620 630 10.1016/0092-8674(93)90244-k 8500161

[B132] Haslam RJ Koide HB Hemmings BA Pleckstrin domain homology Nature 1993 363 309 310 8497315 10.1038/363309b0

[B133] Lemmon MA Ferguson K Signal-dependent membrane targeting by pleckstrin homology (PH) domains Biochem J 2000 350 1 18 10.1042/0264-6021:3500001 10926821 PMC1221219

[B134] Touhara K Inglese J Pitcher JA Shaw G Lefkowitz RJ Binding of G-protein Beta-gamma subunits to pleckstrin homology domains J Biol Chem 1994 269 10217 10220 8144601

[B135] Wang DS Shaw R Winkelmann JC Shaw G Binding of PH domains of beta-adrenergic receptor kinase and beta-spectrin to WD40/beta-transducin repeat containing regions of the beta-subunit of trimeric G-proteins Biochem Biophys Res Commun 1994 203 29 35 10.1006/bbrc.1994.2144 8074669

[B136] Yao L Suzuki H Ozawa K Deng J Lehel C Fukamachi H Anderson WB Kawakami Y Kawakami T Interactions between protein kinase C and pleckstrin homology domains. Inhibition by phosphatidylinositol 4,5-bisphosphate and phorbol 12-myristate 13-acetate J Biol Chem 1997 272 13033 13039 10.1074/jbc.272.20.13033 9148913

[B137] Liu L Makowske M Phosphotyrosine protein of molecular mass 30 kDa binds specifically to the positively charged region of the pleckstrin N-terminal pleckstrin homology domain Biochem J 1999 342 423 430 10.1042/0264-6021:3420423 10455030 PMC1220480

[B138] Morishige M Hashimoto S Ogawa E Toda Y Kotani H Hirose M Wei S Hashimoto A Yamada A Yano H GEP100 links epidermal growth factor receptor signalling to Arf6 activation to induce breast cancer invasion Nat Cell Biol 2008 10 85 92 10.1038/ncb1672 18084281

[B139] Foley J Nickerson NK Nam S Allen KT Gilmore JL Nephew KP Riese DJ II EGFR signalling in breast cancer: Bad to the bone Semin Cell Dev Biol 2010 21 950 961 10.1016/j.semcdb.2010.08.009 PMC2991402 20813200

[B140] Shao H Cheng HY Cook RG Identification and Characterization of Signal Transducer and Activator of Transcription 3 Recruitment sites within the Epidermal Growth Factor Receptor Cancer Res 2003 63 3923 3930 12873986

[B141] Sabe H Hashimoto S Morishige M Ogawa E Hashimoto A Nam JM Miura K Yano H Onodera Y The EGFR-GEP100-Arf6-AMAP1 signalling pathway specific to breast cancer invasion and metastasis Traffic 2009 10 982 993 10.1111/j.1600-0854.2009.00917.x 19416474 PMC2721971

[B142] Valderrama F Ridley AJ Getting invasive with GEP100 and Arf6 Nat Cell Biol 2008 10 16 18 10.1038/ncb0108-16 18172429

[B143] Zhang D Aravind L Identification of novel families and classification of the C2 domain superfamily elucidate the origin and evolution of membrane targeting activities in eukaryotes Gene 2010 469 18 30 10.1016/j.gene.2010.08.006 20713135 PMC2965036

[B144] Cho W Stahelin RV Membrane binding and subcellular targeting of C2 domains Biochim BiophysActa 2006 1761 838 849 10.1016/j.bbalip.2006.06.014 16945584

[B145] Benes CH Wu N Elia AE Dharia T Cantley LC Soltoff SP The C2 domain of PKCdelta is a phosphotyrosine binding domain Cell 2005 121 271 280 10.1016/j.cell.2005.02.019 15851033

[B146] Sondermann H Kuriyan J C2 can do it, too Cell 2005 121 158 160 10.1016/j.cell.2005.04.001 15851022

[B147] Wortmann A He Y Deryugina EI Quigley JP Hooper JD The cell surface glycoprotein CDCP1 in cancer–insights, opportunities, and challenges IUBMB Life 2009 61 723 730 10.1002/iub.198 19514048

[B148] Stahelin RV Kong KF Raha S Tian W Melowic HR Ward KE Murray D Altman A Cho W Protein Kinase Cθ C2 Domain Is a Phosphotyrosine Binding Module That Plays a Key Role in Its Activation J Biol Chem 2012 287 30518 30528 10.1074/jbc.M112.391557 22787157 PMC3436300

[B149] Simister PC Burton NM Brady RL Phosphotyrosine Recognition by the Raf Kinase Inhibitor Protein Forum on Immunopathological Diseases and Therapeutics 2011 2 59 70 10.1615/ForumImmunDisTher.v2.i1.70

[B150] Christofk HR Heiden MGV Wu N Asara JM Cantley LC Pyruvate kinase M2 is a phosphotyrosine binding protein Nature 2008 452 182 189 10.1038/nature06667 18337815

[B151] Edwards A Large-scale structural biology of the human proteome Annu Rev Biochem 2009 78 541 568 10.1146/annurev.biochem.78.070907.103305 19489729

[B152] Wishart MJ Denu JM Williams JA Dixon JE A Single Mutation Converts a Novel Phosphotyrosine Binding Domain into a Dual-specificity Phosphatase J Biol Chem 1995 270 26782 26785 10.1074/jbc.270.45.26782 7592916

[B153] Wishart MJ Dixon JE Gathering STYX: phosphatase-like form predicts functions for unique protein-interaction domains Trends Biochem Sci 1998 23 302 306 10.1016/s0968-0004(98)01241-9 9757831

[B154] Tonks NK Protein tyrosine phosphatases: from genes, to function, to disease Nat Rev Mol Cell Biol 2006 7 833 846 10.1038/nrm2039 17057753

[B155] Almo SC Bonanno JB Sauder JM Emtage S Dilorenzo TP Malashkevich V Wasserman SR Swaminathan S Eswaramoorthy S Agarwal R Structural genomics of protein phosphatases J Struct Funct Genomics 2007 8 121 140 10.1007/s10969-007-9036-1 18058037 PMC4163028

[B156] Tonks NK Pseudophosphatases: grab and hold on Cell 2009 139 464 465 10.1016/j.cell.2009.10.008 19879835

[B157] Hinton SD Myers MP Roggero VR Allison LA Tonks NK The pseudophosphatase MK-STYX interacts with G3BP and decreases stress granule formation Biochem J 2010 427 349 357 10.1042/BJ20091383 20180778 PMC2873733

[B158] Cheng KC Klancer R Singson A Seydoux G Regulation of MBK-2/DYRK by CDK-1 and the pseudophosphatases EGG-4 and EGG-5 during the oocyte-to-embryo transition Cell 2009 139 560 572 10.1016/j.cell.2009.08.047 19879842 PMC2790168

[B159] Gingras MC Zhang YL Kharitidi D Barr AJ Knapp S Tremblay ML Pause A HD-PTP is a catalytically inactive tyrosine phosphatase due to a conserved divergence in its phosphatase domain PLoS One 2009 4 e5105 10.1371/journal.pone.0005105 19340315 PMC2661844

[B160] Li J Clifford Y Danny L Katrina P Shikha B Steven IW Janusz P Christa M Linda R Richard MC PTEN, a Putative Protein Tyrosine Phosphatase Gene Mutated in Human Brain, Breast,and Prostate Cancer Science 1997 275 1943 1948 10.1126/science.275.5308.1943 9072974

[B161] Guan R Dai H Harrison SC Kirchhausen T Structure of the PTEN-like region of auxilin, a detector of clathrin-coated vesicle budding Structure 2010 18 1191 1198 10.1016/j.str.2010.06.016 20826345 PMC2955424

[B162] Lee JO Yang H Georgescu MM Di Cristofano A Maehama T Shi Y Dixon JE Pandolfi P Pavletich NP Crystal Structure of the PTEN Tumor Suppressor: Implications for Its Phosphoinositide Phosphatase Activity and Membrane Association Cell 1999 99 323 334 10.1016/S0092-8674(00)81663-3 10555148

[B163] Yeung K Seitz T Li S Janosch P McFerran B Kaiser C Fee F Katsanakis KD Rose DW Mischak H Sedivy JM Kolch W Suppression of Raf-1 kinase activity and MAP kinase signalling by RKIP Nature 1999 401 173 178 10.1038/43686 10490027

[B164] Park S Rath O Beach S Xiang X Kelly SM Luo Z Kolch W Yeung KC Regulation of RKIP binding to the N-region of the Raf-1 kinase FEBS Lett 2006 580 6405 6413 10.1016/j.febslet.2006.10.054 17097642 PMC1892598

[B165] Rath O Park S Tang HH Banfield MJ Brady RL Lee YC Dignam JD Sedivy JM Kolch W Yeung KC The RKIP (Raf-1 Kinase Inhibitor Protein) conserved pocket binds to the phosphorylated N-region of Raf-1 and inhibits the Raf-1-mediated activated phosphorylation of MEK Cell Signal 2008 20 935 942 10.1016/j.cellsig.2008.01.012 18294816

[B166] Tavel L Jaquillard L Karsisiotis AI Saab F Jouvensal L Brans A Delmas AF Schoentgen F Cadene M Damblon C Ligand Binding Study of Human PEBP1/RKIP: Interaction with Nucleotides and Raf-1 Peptides Evidenced by NMR and Mass Spectrometry PLoS One 2012 7 e36187 10.1371/journal.pone.0036187 22558375 PMC3338619

[B167] Christofk HR Vander Heiden MG Harris MH Ramanathan A Gerszten RE Wei R Fleming MD Schreiber SL Cantley LC The M2 splice isoform of pyruvate kinase is important for cancer metabolism and tumour growth Nature 2008 452 230 234 10.1038/nature06734 18337823

[B168] Mazurek S Pyruvate kinase type M2: a key regulator of the metabolic budget system in tumor cells Int J Biochem Cell Biol 2011 43 969 980 10.1016/j.biocel.2010.02.005 20156581

[B169] Dombrauckas JD Santarsiero BD Mesecar AD Structural Basis for Tumor Pyruvate Kinase M2 Allosteric Regulation and Catalysis Biochemistry 2005 44 9417 9429 10.1021/bi0474923 15996096

[B170] Christofk HR Wu N Cantley LC Asara JM Proteomic screening method for phosphopeptide motif binding proteins using peptide libraries J Proteome Res 2011 10 4158 4164 10.1021/pr200578n 21774532 PMC3174226

[B171] Martin GS The hunting of the Src Nat Rev Mol Cell Biol 2001 2 467 475 10.1038/35073094 11389470

[B172] Sawyer TK Shakespeare WC Wang Y Sundaramoorthi R Huang WS Metcalf CA 3rd Thomas M Lawrence BM Rozamus L Noehre J Protein phosphorylation and signal transduction modulation: chemistry perspectives for small-molecule drug discovery Med Chem 2005 1 293 319 10.2174/1573406053765486 16787325

[B173] Lappalainen I Thusberg J Shen B Vihinen M Genome wide analysis of pathogenic SH2 domain mutations Proteins 2008 72 779 792 10.1002/prot.21970 18260110

[B174] Hong Y Chalkia D Ko KD Bhardwaj G Chang GS van Rossum DB Patterson RL Phylogenetic Profiles Reveal Structural and Functional Determinants of Lipid-binding J Proteomics Bioinform 2009 2 139 149 10.4172/jpb.1000071 19946567 PMC2782862

[B175] Koskela HLM Eldfors S Somatic STAT3 Mutations in Large Granular Lymphocytic Leukemia N Engl J Med 2012 366 1905 1913 10.1056/NEJMoa1114885 22591296 PMC3693860

[B176] Giacomelli M Tamassia N Moratto D Bertolini P Ricci G Bertulli C Plebani A Cassatella M Bazzoni F Badolato R SH2-domain mutations in STAT3 in hyper-IgE syndrome patients result in impairment of IL-10 function Eur J Immunol 2011 41 3075 3084 10.1002/eji.201141721 21792878

[B177] Machida K Mayer BJ The SH2 domain: versatile signalling module and pharmaceutical target Biochim Biophys Acta 2005 1747 1 25 10.1016/j.bbapap.2004.10.005 15680235

[B178] Lu XL Cao X Liu XY Jiao BH Recent progress of Src SH2 and SH3 inhibitors as anticancer agents Curr Med Chem 2010 17 1117 1124 10.2174/092986710790827861 20158477

[B179] Grebien F Hantschel O Wojcik J Kaupe I Kovacic B Wyrzucki AM Gish GD Cerny-Reiterer S Koide A Beug H Pawson T Valent P Koide S Superti-Furga G Targeting the SH2-Kinase Interface in Bcr-Abl Inhibits Leukemogenesis Cell 2011 147 306 319 10.1016/j.cell.2011.08.046 22000011 PMC3202669

[B180] Bloom L Calabro V FN3: a new protein scaffold reaches the clinic Drug Discov Today 2009 14 949 955 10.1016/j.drudis.2009.06.007 19576999

[B181] Wojcik J Hantschel O Grebien F Kaupe I Bennett KL Barkinge J Jones RB Koide A Superti-Furga G Koide S A potent and highly specific FN3 monobody inhibitor of the Abl SH2 domain Nat Struct Mol Biol 2010 17 519 527 10.1038/nsmb.1793 20357770 PMC2926940

[B182] Jadwin JA Ogiue-Ikeda M Machida K The application of modular protein domains in proteomics FEBS Lett 2012 586 2586 10.1016/j.febslet.2012.04.019 22710164 PMC3413744

[B183] Machida K Mayer BJ Nollau P Profiling the global tyrosine phosphorylation state Mol Cell Proteomics 2003 2 215 233 12754303 10.1074/mcp.R300002-MCP200

[B184] Dierck K Machida K Voigt A Thimm J Horstmann M Fiedler W Mayer BJ Nollau P Quantitative multiplexed profiling of cellular signalling networks using phosphotyrosine-specific DNA-tagged SH2 domains Nat Methods 2006 3 737 744 10.1038/nmeth917 16929320

[B185] Machida K Thompson CM Dierck K Jablonowski K Karkkainen S Liu B Zhang H Nash PD Newman DK Nollau P High-throughput phosphotyrosine profiling using SH2 domains Mol Cell 2007 26 899 915 10.1016/j.molcel.2007.05.031 17588523

[B186] Dierck K Machida K Mayer BJ Nollau P Profiling the tyrosine phosphorylation state using SH2 domains Methods Mol Biol 2009 527 131 155 10.1007/978-1-60327-834-8_11 19241011

[B187] Machida K Eschrich S Li J Bai Y Koomen J Mayer BJ Haura EB Characterizing tyrosine phosphorylation signalling in lung cancer using SH2 profiling PLoS One 2010 5 e13470 10.1371/journal.pone.0013470 20976048 PMC2957407

[B188] Nagahara H Vocero-Akbani AM Transduction of full-length TAT fusion proteins into mammalian cells: TAT-p27Kip1 induces cell migration Nat Med 1998 4 1449 1452 10.1038/4042 9846587

[B189] Katterle Y Brandt BH Dowdy SF Niggemann B Zanker KS Dittmar T Antitumour effects of PLC-gamma1-(SH2)2-TAT fusion proteins on EGFR/c-erbB-2-positive breast cancer cells Br J Cancer 2004 90 230 235 10.1038/sj.bjc.6601506 14710234 PMC2395298

[B190] Saito Y Furukawa T Arano Y Fujibayashi Y Saga T Fusion protein based on Grb2-SH2 domain for cancer therapy Biochem Biophys Res Commun 2010 399 262 267 10.1016/j.bbrc.2010.07.066 20655296

[B191] Saito Y Furukawa T Arano Y Fujibayashi Y Saga T Basic study on SH2 domain of Grb2 as a molecular probe for detection of RTK activation Int J Oncol 2010 37 281 287 20596655 10.3892/ijo_00000676

[B192] Wang Y Botvinick EL Visualizing the mechanicalactivation of Src Nature 2005 434 1040 1045 10.1038/nature03469 15846350

[B193] Antczak C Bermingham A Domain-Based Biosensor Assay to Screen for Epidermal Growth Factor Receptor Modulators in Live Cells Assay Drug Dev Technol 2012 10 24 36 10.1089/adt.2011.423 22280060 PMC3277729

[B194] Amit I Wides R Yarden Y Evolvable signalling networks of receptor tyrosine kinases: relevance of robustness to malignancy and to cancer therapy Mol Syst Biol 2007 3 151 18059446 10.1038/msb4100195 PMC2174628

[B195] Kitano H Cancer as a robust system:implications for anticancer therapy Nat Rev Cancer 2004 4 227 235 10.1038/nrc1300 14993904

[B196] Miller WT Tyrosine kinase signalling and the emergence of multicellularity Biochim Biophys Acta 2012 1823 1053 1057 10.1016/j.bbamcr.2012.03.009 22480439 PMC3358447

[B197] Suga H Dacre M de Mendoza A Shalchian-Tabrizi K Manning G Ruiz-Trillo I Genomic survey of premetazoans shows deep conservation of cytoplasmic tyrosine kinases and multiple radiations of receptor tyrosine kinases Sci Signal 2012 5 ra35 10.1126/scisignal.2002733 22550341

[B198] Jadeau F Grangeasse C Shi L Mijakovic I Deleage G Combet C BYKdb: the Bacterial protein tYrosine Kinase database Nucleic Acids Res 2012 40 D321 D324 10.1093/nar/gkr915 22080550 PMC3245071

[B199] Cozzone AJ Bacterial tyrosine kinases: novel targets for antibacterial therapy? Trends Microbiol 2009 17 536 543 10.1016/j.tim.2009.09.005 19853456

[B200] Gruenheid S Enteropathogenic E. coli Tir binds Nck to initiate actin pedestal formation in host cells Nat Cell Biol 2001 3 856 859 10.1038/ncb0901-856 11533668

[B201] Backert S Tegtmeyer N Selbach M The Versatility of Helicobacter pylori CagA Effector Protein Functions: The Master Key Hypothesis Helicobacter 2010 15 163 176 10.1111/j.1523-5378.2010.00759.x 20557357

[B202] Selbach M Paul FE Brandt S Guye P Daumke O Backert S Dehio C Mann M Host cell interactome of tyrosine-phosphorylated bacterial proteins Cell Host Microbe 2009 5 397 403 10.1016/j.chom.2009.03.004 19380118

[B203] Blasutig IM New LA Thanabalasuriar A Dayarathna TK Goudreault M Quaggin SE Li SS Gruenheid S Jones N Pawson T Phosphorylated YDXV motifs and Nck SH2/SH3 adaptors act cooperatively to induce actin reorganization Mol Cell Biol 2008 28 2035 2046 10.1128/MCB.01770-07 18212058 PMC2268406

[B204] Frischknecht F Moreau V Actin-based motility of vaccinia virus mimics receptor tyrosine kinase signalling Nature 1999 401 926 928 10.1038/44860 10553910

[B205] Weisswange I Newsome TP Schleich S Way M The rate of N-WASP exchange limits the extent of ARP2/3-complex-dependent actin-based motility Nature 2009 458 87 91 10.1038/nature07773 19262673

